# Strigolactones modulate stem length and diameter of cherry rootstocks through interaction with other hormone signaling pathways

**DOI:** 10.3389/fpls.2023.1092654

**Published:** 2023-02-09

**Authors:** Xunju Liu, Yan Xu, Wanxia Sun, Jiyuan Wang, Yixin Gao, Lei Wang, Wenping Xu, Shiping Wang, Songtao Jiu, Caixi Zhang

**Affiliations:** Department of Plant Science, School of Agriculture and Biology, Shanghai Jiao Tong University, Shanghai, China

**Keywords:** cherry rootstocks, strigolactones, stem growth and development, transcriptomics, differentially expressed genes

## Abstract

Stem growth and development has considerable effects on plant architecture and yield performance. Strigolactones (SLs) modulate shoot branching and root architecture in plants. However, the molecular mechanisms underlying SLs regulate cherry rootstocks stem growth and development remain unclear. Our studies showed that the synthetic SL analog rac-GR24 and the biosynthetic inhibitor TIS108 affected stem length and diameter, aboveground weight, and chlorophyll content. The stem length of cherry rootstocks following TIS108 treatment reached a maximum value of 6.97 cm, which was much higher than that following rac-GR24 treatments at 30 days after treatment. Stem paraffin section showed that SLs affected cell size. A total of 1936, 743, and 1656 differentially expressed genes (DEGs) were observed in stems treated with 10 μM rac-GR24, 0.1 μM rac-GR24, and 10 μM TIS108, respectively. RNA-seq results highlighted several DEGs, including *CKX*, *LOG*, *YUCCA*, *AUX*, and *EXP*, which play vital roles in stem growth and development. UPLC-3Q-MS analysis revealed that SL analogs and inhibitors affected the levels of several hormones in the stems. The endogenous GA_3_ content of stems increased significantly with 0.1 μM rac-GR24 or 10 μM TIS108 treatment, which is consistent with changes in the stem length following the same treatments. This study demonstrated that SLs affected stem growth of cherry rootstocks by changing other endogenous hormone levels. These results provide a solid theoretical basis for using SLs to modulate plant height and achieve sweet cherry dwarfing and high-density cultivation.

## 1 Introduction

The global popularity of sweet cherry (*Prunus avium* L.) among consumers can be attributed to its bright color, rich aroma, and high nutritional value (Hayaloglu and Demir, 2016; Ceccarelli et al., 2020). High-density and dwarfing cultivation, in addition to mechanized orchard management, are emerging trends in sweet cherry cultivation (Acero et al., 2019). Because sweet cherry is highly heterozygous, similar to many fruit species of the *Rosaceae* family, it is rarely propagated from seeds. Instead, scions are usually grafted onto rootstocks of the same or another *Prunus* species to propagate preferable sweet cherry cultivars (Webster, 2001). Rootstocks affect numerous horticultural characteristics such as plant height, yield, and the tolerance to biotic and abiotic stress (Isaakadis et al., 2004; Jensen et al., 2010; Liang et al., 2020). Krymsk^®^5 (VLS-2) is a semi-dwarf cherry rootstocks variety originating in Russia, and is a hybrid of *P. fruticosa* and *P. lannesiana*. It is more durable than Mazard because it adapts to different types of soil (Tsafouros and Roussos, 2019). Dwarfing and intensive planting are the general trends in agricultural production. Dwarfing is an essential indicator of cherry rootstocks breeding and largely determines the mechanization level of cherry orchard management. Thus, investigating the molecular mechanisms of stem growth and development in cherry rootstocks is crucial for manipulating plant height to achieve cherry dwarfing and high-density cultivation.

Several phytohormones such as gibberellins (GAs), auxin, brassinosteroid (BR), and cytokinin (CTK) are involved in the regulation of stem growth and development in plants (Ji and Yang, 2002; Davies, 2010). GAs regulate plant height and organ size by stimulating cell division and elongation (Ayano et al., 2014; Zhou and Underhill, 2016; Marzec, 2017). Previous studies have revealed that the alteration of GA content or signaling result in dwarf or slender phenotype (Mitchum et al., 2006; Zhang et al., 2016; Nagai et al., 2020). Plant-stature-related receptor-like Kinanse2 (PSRK2) is a critical component of the GA signaling pathway that negatively regualte GA responses to modulate stem elongation (Li et al., 2018). Previous studies have shown that GA_3_ in sorghum (Sorghum bicolor) increas cell length, the number of cells spanning internode (Yu et al., 2021). In addition, BR directly regulates the plant height and leaf angle in rice. The absence of BR or a decrease in BR perception leads to a dwarfing phenotype in rice, indicating that BR specifically regulates plant height of rice (Sun et al., 2015; Liu et al., 2016; Castorina and Consonni, 2020). The length of rose shoots treated with the cytokinin analog thidiazuron (TDZ) was 50% lower that of the control, while the stem diameter was 40% lower than that of control (Kucharska and Orlikowska, 2009). After TDZ treatment, the expression of GA synthesis genes decreased considerably and the expression of GA decomposition genes increased marginally, indicating that TDZ regulate the stem development of roses by regulating the metabolism of bioactive GA (Çelikel et al., 2021).

Strigolactones (SLs) are associated with numerous developmental processes and environmental responses, and are mainly synthesized in the roots before being transported to aboveground (Ma et al., 2020; Mitra et al., 2021; Xu et al., 2021). In previous reports, rac-GR24 and TIS108 are widely used as synthetic SL analog and biosynthesis inhibitor (Ito et al., 2011; Zwanenburg and Blanco-Ania, 2018; Barbier et al., 2019). SLs play an important role in modulating shoot branching in various plants (Gomez-Roldan et al., 2008; Xu et al., 2015; Yoneyama and Brewer, 2021), and have been suggested to inhibit bud outgrowth (Zhuo et al., 2021). A previous report revealed complex interactions among cytokinins, SLs, and auxins in the regulation of shoot branching in *Oryza sativa* (Zha et al., 2019). However, further investigation is required to determine the mechanisms by which SLs regulate stem growth and development, and their interaction with other hormones.

Gene expression analysis is one of the most effective methods for elucidating plant growth and development processes. The sequencing of sweet cherry genome enables the study of gene expression patterns under different conditions. To elucidate the mechanism by which SLs regulate stem growth and development in cherry rootstocks, endogenous hormone levels of stem under SL or TIS108 treatment were first measured in this study. Furthermore, transcriptome analysis was performed to screen for candidate genes related to hormones signaling and stem growth and development. This study aimed to reveal the molecular mechanism by which SLs affect stem growth and development of cherry rootstocks to provide a strong theoretical basis for using SLs to modulate plant height of sweet cherry. Ultimately, our findings could be applied to sweet cherry dwarfing and high-density cultivation.

## 2 Materials and methods

### 2.1 Plant materials, hormone treatments, and growth conditions

Woody plant medium (WPM) containing 0.8% (w/v) agar, 3% (w/v) sucrose, and 0.5 mg/L indole-3-butyric acid was used for the propagation of cherry rootstocks Krymsk^®^5. Plant seedlings were grown on tissue cultures at 23°C for 8 h in the dark and 16 h under artificial light. Before sterilization, the pH of the medium was adjusted to suitable pH of 5.8. Before rac-GR24 or TIS108 treatment, stem tips of approximately 3–4 cm were subcultured for 5 d in WPM to promote the formation of new roots. The tips were then removed, rinsed with sterile water 6–8 times, and transferred to fresh WPM containing 0.8% (w/v) agar, 3% (w/v) sucrose, and treated with 0.1 μM rac-GR24 (S1), 10 μM rac-GR24 (S2), or 10 μM TIS108 (T) (Xu et al., 2021; Jiu et al., 2021). The control group was placed in WPM containing 0.8% (w/v) agar and 3% (w/v) sucrose, without phytohormones. Thirty-six cherry rootstocks plantlets were used in each treatment and evenly divided into three harvest dates. Four plantlets were grouped together and considered a replicate. There were three replicates per treatment for each harvest date. Stem length was recorded at 5, 10, and 30 days after treatment (DAT). The stem diameter and fresh weight of the aboveground part were measured at 30 DAT. The third and fourth leaves above the root–stem junction were used to measure the chlorophyll content using a portable chlorophyll meter (SPAD-502; Minolta, Osaka, Japan) at 30 DAT. In addition, we recorded the number of internodes and the length of the third and fourth internodes at 30 DAT. In our study, stem length and diameter showed the most significant differences at 30 DAT. Thus, we selected these samples from this period for subsequent analysis. We collected stem samples at 30 DAT, froze them in liquid nitrogen, and stored them at −80°C until further use.

### 2.2 Paraffin section detection

At 30 DAT, stem segments about 2–3 cm from the top of the stem tip were collected and examined in longitudinal sections at the widest part of the stem. In addition, we counted the number, length and width of cells in stems of cherry rootstocks treated with different treatments. The preparation of paraffin sections followed the method of Gao et al. (2021). The samples were observed, photographed, and analyzed using an Olympus BX61 microscope (Olympus, Tokyo, Japan).

### 2.3 Detection of endogenous hormone levels

All experiments were conducted using an ultra-high-performance liquid chromatography system (Waters, Milford, MA, USA) equipped with an electrospray ionization source and a 5500 Qtrap Mass Spectrometer (MS) (AB SCIEX, Foster City, CA, USA). Analyst 1.6.2 software (AB SCIEX, Foster City, CA, USA) was used to collect and process the data. Plant hormones were isolated using a BEH C18 column (130 Å, 1.7 μm, 2.1 × 100 μm). The contents of GAs, 3-indoleacetic acid (IAA), abscisic acid (ABA), and SLs were determined based on previous reports (Rial et al., 2018; Xin et al., 2020; Jiu et al., 2021). We purchased internal isotope standards of IAA and ABA from OlChemIm (Olomouc, Czech Republic), and GA_3_, GA_4,_ and GA_7_ from ANPEL Laboratory Technologies Inc. (Shanghai, China). Standard substances of (±)2’-epi-5-deoxystrigol (DS) and strigol were purchased from OlChemIm (Olomouc, Czech Republic). TIS108 and rac-GR24 were purchased from Beijing Daqin Science Co. Ltd. (Beijing, China). We used pg/g fresh weight (Fw) to describe the content of (±)2’-epi-5-DS and strigol. Furthermore, the ABA, IAA, GA_3_, GA_4_, and GA_7_ contents were expressed as ng/g Fw.

### 2.4 RNA isolation and Illumina sequencing

Triazole reagent (Takara, Dalian, China) was used to extract total RNA from the stem samples according to the manufacturer’s instructions. Genomic DNA was removed using RNase-free DNase I (NEB, Beijing, China) (Jiu et al., 2022). RNA concentration was measured using an Agilent 2100 biological analyzer (Santa Clara, California, USA). Subsequently, RNA quality was assessed by agarose gel electrophoresis as previously described (Jiu et al., 2020). Analysis included RNA integrity number ≥ 7.5, A260/A280 ratio between 1.9 and 2.1, and RNA 28S/18S ratio > 1. Using Dynal beads Oligo (dT) _25_ (Dynal Biotech, Oslo, Norway), poly(A)^+^ RNA was separated from the total RNA to construct Illumina sequencing libraries according to the manufacturer’s guidelines. The cDNA libraries was sequenced by Shanghai Personal Biotechnology Co. Ltd. (Shanghai, China).

### 2.5 Read mapping and differential expression analysis

Raw reads from the NovaSeq platform were filtered out adapters, low-quality reads, and shorter reads. HISAT2 (http://ccb.jhu.edu/software/hisat2/index.shtml) was used to align the clean reads against the sweet cherry genome (https://www.rosaceae.org/species/prunus_avium/genome_v1.0.a1) (Kim et al., 2015). We used only read fragments with unique mapping for subsequent analysis. Normalized expression levels were determined using fragments per kilobase of exon per million fragments mapped (FPKM). Genes with a *p*-value < 0.05 and the lowest two-fold difference in expression (|log_2_Fold change |> 1) were defined as significantly differentially expressed. Volcano and MA maps of the DEGs were constructed using ggplot2 (https://cran.rproject.org/web/packages/ggplot2/index.html). A two-way clustering analysis was conducted for DEGs of all comparison groups using the heatmap R package (https://www.datanovia.com/en/lessons/heatmap-in-r-static-and-interactive-visualization/).

### 2.6 Gene annotation and enrichment analysis

All DEGs were annotated based on a Basic Local Alignment Search Tool (BLAST) search and searched against protein databases. All genes were mapped to Gene Ontology (GO) terms in the GO database and to Kyoto Encyclopedia of Genes and Genomes (KEGG) orthologs in the KEGG pathway database. GO enrichment analysis of DEGs was performed using topGO (*p* < 0.05) as previously described (Tian et al., 2022). The statistical enrichment of DEGs was tested using ClusterProfiler (3.4.4) software (Yu et al., 2012), and a *p*-value <0.05 was considered significantly enriched in KEGG pathways.

### 2.7 Quantitative real-time polymerase chain reaction (qRT-PCR) validation

A CFX Connect Real-Time System (Bio-Rad, Munchen, Germany) was used for qRT-PCR. The 10 µL PCR mixture included 1 μL of cDNA, 5 μL of 2× TB Green II mix, 0.5 µL each of 10 µM reverse primer and 10 µM forward primer, and 3.0 μL of ddH_2_O. Parameters reported by Jia et al. (2016) were used for the qRT-PCR assay. Three technical replicates were performed for each sample. The 2^–ΔΔCT^ method (Livak and Schmittgen, 2001) was used to calculate the relative expression levels of each gene. As an internal reference control, *PavActin* was used to normalize gene expression levels (Jiu et al., 2019; Wang et al., 2021), and the primer sequences used in the qRT-PCR assay are listed in **Table S1**.

### 2.8 Statistical analysis

The experiment was conducted using a completely randomized design with three replicates. Data analysis was performed using the SAS software package (version 9.2, SAS Institute Inc. Cary, NC, USA). A one-way analysis of variance was used to determine statistical differences between groups. For the results of the analysis of stem length and diameter, aboveground weight, chlorophyll, phytohormone content, and qRT-PCR assay, data are presented as the mean ± standard deviation (SD) of the three replicates.

## 3 Results

### 3.1 Effect of SL analog and biosynthetic inhibitor on the aboveground parts of cherry rootstocks

**Figure 1:**
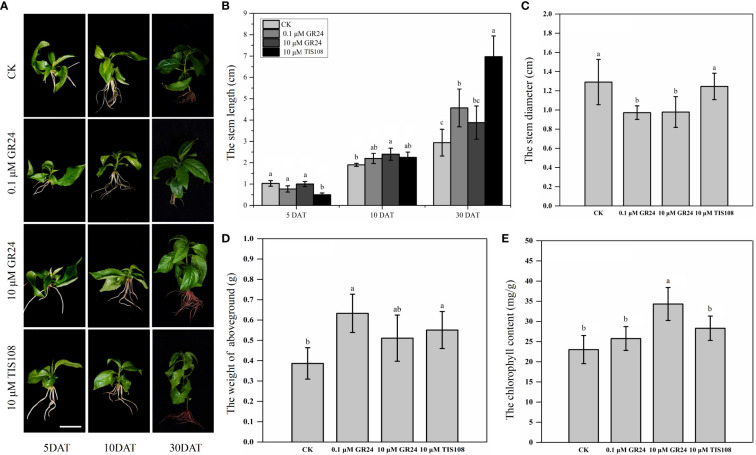
Growth characteristics of cherry rootstocks treated with exogenous rac-GR24 and TIS108 treatments. **(A)** Phenotypic characteristics of aboveground part in cherry rootstocks treated with exogenous rac-GR24 or TIS108. Bar = 2 cm. **(B)** Stem length of cherry rootstocks treated with exogenous rac-GR24 and TIS108 at 5, 10, and 30 days after treatment (DAT). Stem diameter **(C)**, fresh weight (Fw) of aboveground **(D)**, and chlorophyll content **(E)** of cherry rootstocks treated with exogenous rac-GR24 and TIS108 at 30 DAT. The data represent means ± standard deviation (SD) of three replicates. Statistical significance was determined by one-way analysis of variance; significant differences among means (LSD, *p* < 0.05) are indicated by different lowercase letters.

**Figure 2:**
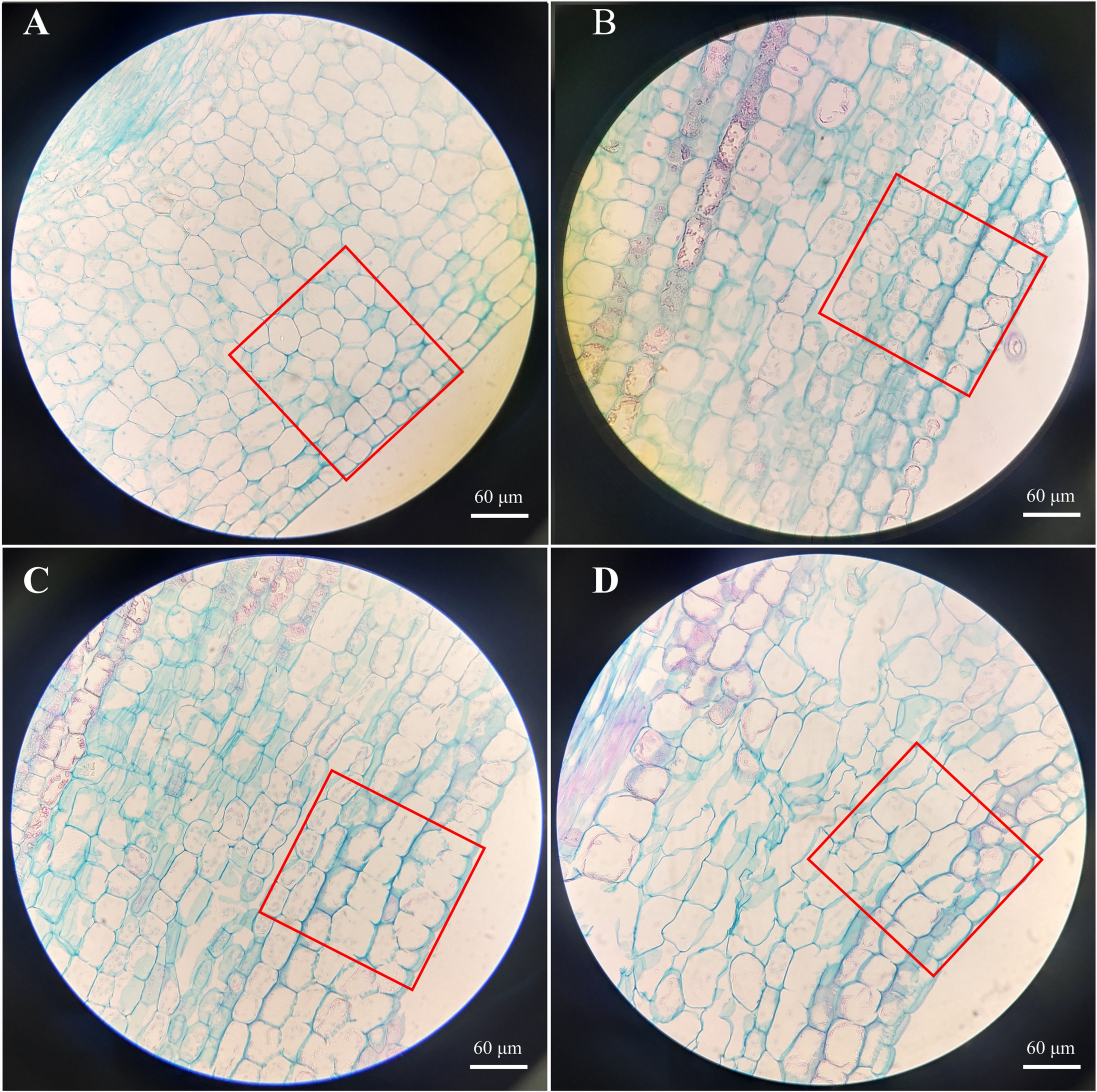
Stem paraffin section of cherry rootstocks at control group **(A)** and 30 d after 0.1 μM rac-GR24 **(B)**, 10 μM rac-GR24 **(C)**, and 10 μM TIS108 **(D)** treatments. Bar = 60 μm.

The phenotypic traits of the aboveground parts were recorded to determine the physiological effects of SLs on the aboveground growth and development of cherry rootstocks (**Figure 1** and **Table S2**). Stem length and diameter, Fw of aboveground parts, and chlorophyll content of the plantlets treated with rac-GR24 or TIS108 differed from those of the control group (**Figures 1A–E**). The Fw of the aboveground parts following S1, S2, and T treatments increased by 64%, 32%, and 43%, respectively. In contrast, the Fw of the aboveground part following S2 treatment did not differ significantly from that of the control plantlets (**Figure 1D**). This indicates that 0.1 μM rac-GR24 treatment acts as a positive regulator for the Fw of the aboveground part at 30 DAT. As shown in **Figure 1E**, the chlorophyll content in S2 treatment exhibited a maximum value of 34.31 mg/g, which was higher than those of the other two treatments and control groups. At 10 DAT, the stem length increased slightly following S1 or T treatment; however, it did not differ significantly with that of the control. At 30 DAT, the stem length of plantlets in the S1 and T treatments increased by 54% and 137%, respectively, compared with that of the control group, reaching 4.57 cm and 6.97 cm, respectively. Meanwhile, the stem length following S2 treatment showed no difference compared with that of the control group at 30 DAT (**Figure 1B**). The stem diameters of S1- and S2-treated plantlets were consistently lower than those of the control group, indicating that SLs contributed to inhibiting stem thickening (**Figure 1C**). In addition, as shown in **Figure S1A–C**, the difference of number of internodes is not significant after S1, S2, and T treatments at 30 DAT. However, after different treatments, the internode length of the third and fourth internodes differed significantly, which may indicate that SLs change the plant height by affecting the internode length of cherry rootstocks. These results show that the exogenous application of SL analog and inhibitor greatly affected the stem growth and development of cherry rootstocks. Additionally, we observed the cellular structure of the stems using paraffin sections (**Figure 2**). At 30 DAT, we counted the cell number and sizes in the longitudinal section of cherry rootstocks. The data showed that under the same area, the number of cells in CK, S1, S2, and T treatments were about 41, 30, 32 and 26, respectively. The cell sizes in T treatment were significantly greater than those in the control group (**Figures S1D–F**). These results suggest that SLs inhibitor could affect the cortical cell size.

### 3.2 SL analog and inhibitor alter endogenous hormone contents of plantlet stems

**Figure 3:**
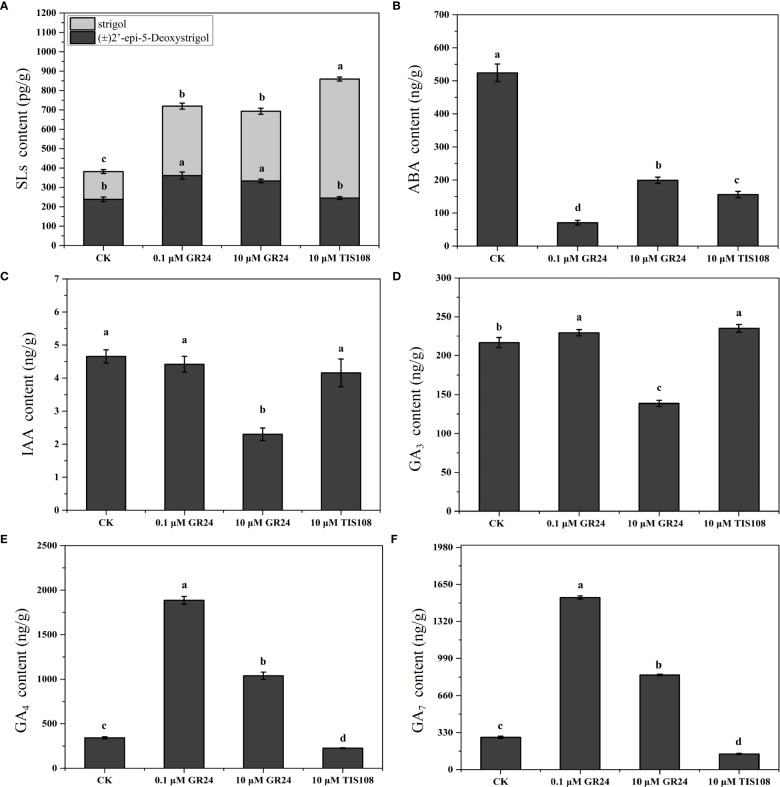
Endogenous hormone levels of cherry rootstocks treated with rac-GR24 or TIS108 at 30 days after treatment (DAT). Major six classes of hormone compounds of the young stems in cherry rootstocks, treated with rac-GR24 or TIS108 at 30 days after treatment (DAT). Data are shown as mean ± standard deviation (SD) of three replicates. Statistical significance determined by one-way analysis of variance (ANOVA); significant differences among means (LSD, *p* < 0.05) are indicated with different lowercase letters.

To determine the differences in the stem of cherry rootstocks treated with TIS108 and rac-GR24, the contents of IAA, gibberellin 3 (GA_3_), SLs, gibberellin 4 (GA_4_), ABA, and gibberellin 7 (GA_7_) were detected at 30 DAT (**Figure 3** and **Table S3**). We detected two types of SLs with different molecular weights, (±)2’-epi-5-DS and strigol. The (±)2’-epi-5-DS content in the stem increased considerably under S1 and S2 treatments; however, under T treatment, no significant difference was observed with that of the control group (**Figure 3A**). Compared with the control group, the (±)2’-epi-5-DS content of stems following both S1 and S2 treatments increased 52% and 40%, reaching 360.89 and 332.96 pg/g, respectively (**Figure 3A**). The IAA content of the stems decreased considerably following S2 treatment, whereas it exhibited no apparent differences following S1 and T treatments when compared with the control group (**Figure 3C**). The GA_3_ content of the stems decreased considerably following S2 treatment compared with the control group. Nevertheless, it increased following S1 and T treatments. This shows that different concentrations of rac-GR24 have different effects on the content of endogenous GA_3_ (**Figure 3D**). The contents of GA_4_ and GA_7_ under S1 and S2 treatments were markedly higher than those of the control group. In comparison, the opposite trend was observed under T treatment (**Figures 3E, F**). Additionally, the data showed that the contents of GA_4_ and GA_7_ of stem in low concentration (0.1 μM) of rac-GR24 treatment were higher than those in high concentration (10 μM) of rac-GR24 treatment. The results showed that SL may regulate the growth and development of cherry rootstocks stem by changing other endogenous hormone levels.

### 3.3 Significant DEGs found at 30 days after S1, S2, and T treatments

**Figure 4:**
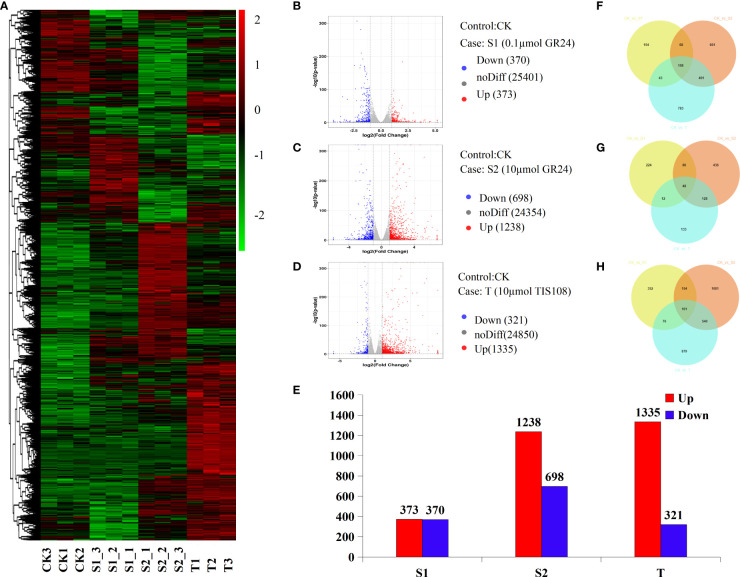
Transcriptional changes in cherry rootstocks stems at 30 days after treatment (DAT) with GR24 or TIS108. Expression profiles of genes in cherry rootstocks plantlets treated with 0.1 μM rac-GR24 (S1), 10 μM rac-GR24 (S2), and 10 μM TIS108 (T) are shown by the heatmap **(A)**. Volcano map of S1 **(B)**, S2 **(C)**, and T **(D)** groups. Red and green histograms represent the log_2_fold-change values of up- and down-regulated genes, respectively. The Gray scatter plot shows the log_2_fold-change values of the non-differentially expressed genes. **(E)** Number of up regulated and down regulated genes in rac-GR24 or TIS108 treatments. Venn diagrams show the proportions of all **(F)**, up regulated **(G)**, and down regulated **(H)** DEGs in three treatments. CK, control group.

In this study, 513,057,306 raw reads were produced from 12 libraries and were uploaded to the NCBI sequence Read Archive database (PRJNA752465). We obtained 36,765,296–41,536,896 clean reads, with an average of 39,466,959. Over 94.9% of clean reads were uniquely mapped to the cherry genome (**Tables S4, S5**). Pearson’s correlation analysis showed a strong correlation between the three biological replicates (**Figure S2**). This indicates that the accuracy and quality of the RNA-seq data met the requirements of subsequent analysis. To identify candidate genes in response to rac-GR24 and TIS108 treatments, we focused on DEGs with significant expression levels between the treatment and the control group. The global gene expression profile is shown using a circle (**Figure S3**) and a heat map (**Figure 4A**). The results showed that 373, 1238, and 1335 genes were upregulated, while 370, 698, and 321 genes were downregulated under S1, S2, and T treatments, respectively, when compared with the control group (**Figures 4B–E**). As shown in **Figures 4F–H**, the stems of cherry rootstocks treated with TIS108 or rac-GR24 contain a greater number of unique and mutual DEGs in the Venn diagram.

### 3.4 The analysis of GO enrichment and KEGG pathway

Comparing the libraries of the three treatments using GO analysis revealed the biological functions of DEGs. As shown in **Figure S4**, under S1 treatment, apoplasts were the top GO terms in the cellular component (CC). DNA-binding transcription factor activity was the most prominent GO term in molecular function (MF), followed by transcription regulator activity. Cellular carbohydrate metabolic process and oxidation-reduction processes were the two largest GO terms in biological process (BP). Under S2 treatment, the membrane was the top GO term in the CC. The catalytic and oxidoreductase activities were the most prominent GO terms in MF. The oxidation-reduction process and response to biotic stimuli were the two largest GO terms in BP (**Figure S5**). Under T treatment, the top GO term in the CC was membrane, and the two largest GO terms in the MF were tetrapyrrole binding and oxidoreductase activity. The two largest GO terms were a response to biotic stimuli and an oxidation-reduction process in BP (**Figure S6**).

The results showed that 115 of the 2,877 DEGs between S1 and control (CK) groups were enriched in 50 pathways (**Figure S7**, **Table S6**), 385 of the 4,467 DEGs between S2 and CK groups were enriched in 92 pathways (**Figure S8**, **Table S7**), and 276 of the 3,999 DEGs between T and CK groups were enriched in 75 pathways (**Figure S9**, **Table S8**). Comprehensive functional enrichment analysis revealed that the citrate cycle (pavi00020), phenylalanine, tyrosine, tryptophan biosynthesis (pavi00400), and RNA degradation (pavi03018) were only enriched in CK *vs*. S2, suggesting that treatment with 10 μM rac-GR24 may expedite plant growth and development. Only CK *vs*. T recorded enriched fatty acid biosynthesis (pavi00061) and lysine biosynthesis (pavi00300), suggesting that TIS108 may activate these pathways. These annotations provide many essential indicators for investigating the specific biological processes and physiological functions involved in the SL-induced growth and development process in cherry rootstocks stems.

### 3.5 The DEGs related to hormone metabolic and signal transduction pathways

The expression levels of DEGs involved in the hormone pathway were studied to understand the changes of hormone-related genes in cherry rootstocks treated with exogenous TIS108 and rac-GR24. Here, DEGs related to hormone metabolism pathways were classified into five groups. Following are the findings observed in each group.

#### 3.5.1 Expression profile of SL- or BR-related genes

**Figure 5:**
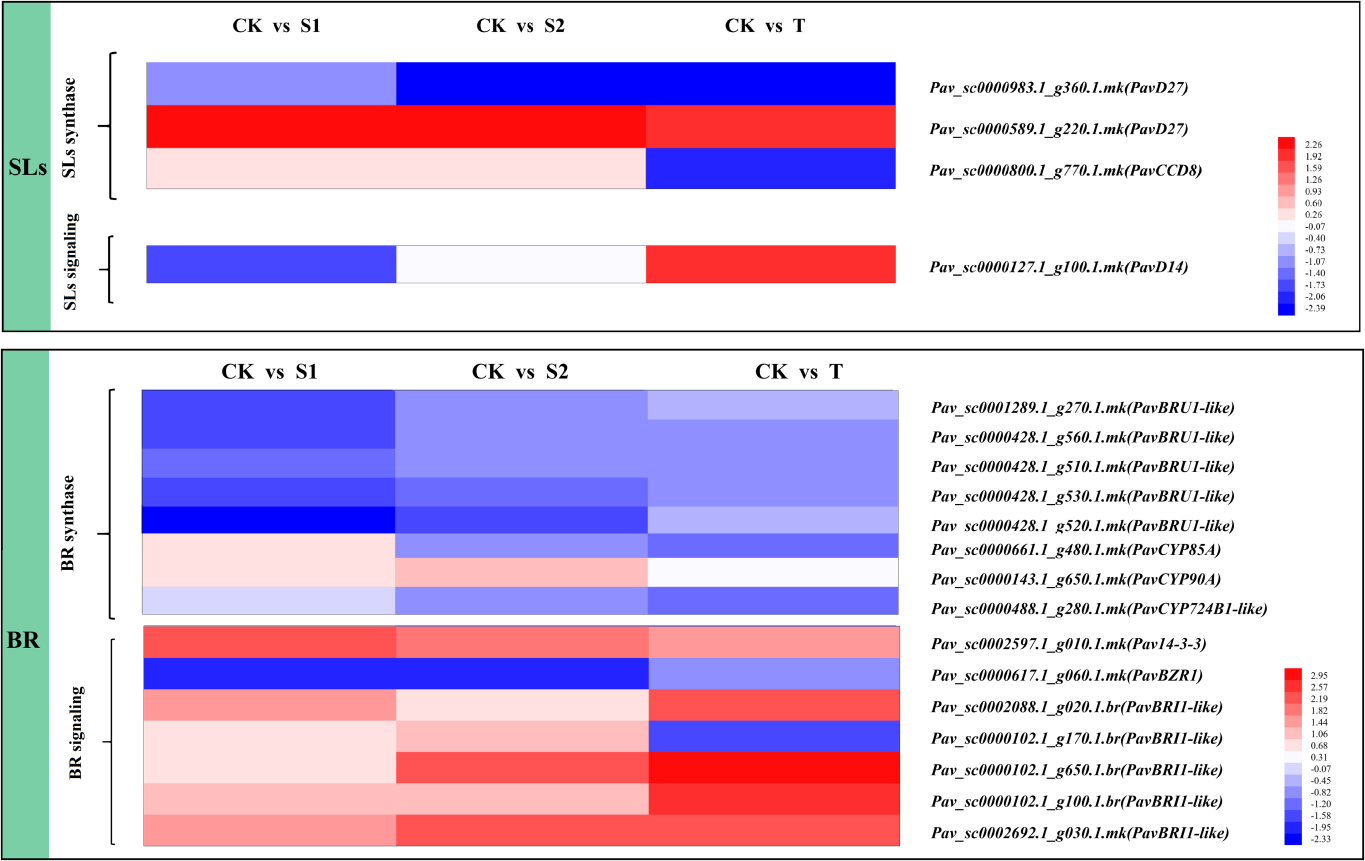
Expression profiles of differentially expressed genes (DEGs) related to strigolactones (SLs) and brassinosteroid (BR) synthesis, and signal transduction pathways. The scale of the color intensity is in the lower-right quarter of the heatmap, representing the log_2_fold-change values. Fold-change refers to the ratio of gene expression levels in cherry rootstocks stems between the control (CK) and treatment (S1/S2/T).

The expression levels of DEGs involved in SL or BR biosynthesis and signaling pathways are shown in **Figure 5** and **Tables S9, S10**. The SL-related genes included beta-carotene isomerase D27 homolog (*PavD27*), SL esterase D14 homolog (*PavD14*), and carotenoid cleavage dioxygenase 8 (*PavCCD8*). *PavD14* (*Pav_sc0000127.1_g100.1.mk*), which acts as a receptor of SL, exhibited different expression patterns under three treatments. The transcript level of *PavD14* (*Pav_sc0000127.1_g100.1.mk*) was notably upregulated under T treatment and downregulated under S1 treatment. *PavCCD8* (*Pav_sc0000800.1_g770.1.mk*) was inhibited by T treatment; however, no significant changes were observed under S1 and S2 treatments. The BR-related genes BRI1 kinase inhibitor 1 (*PavBKI1*) and BR-regulated protein (*PavBRU1-like*) were found in the DEGs files of the transcriptome. The data showed that five transcripts (*Pav_sc0001289.1_g270.1.mk*, *Pav_sc0000428.1_g560.1.mk*, *Pav_sc0000428.1_g510.1.mk*, *Pav_sc0000428.1_g530.1.mk*, and *Pav_sc0000428.1_g520.1.mk*) of *PavBRU1-like* were significantly inhibited by S1 treatment. Two transcripts (*Pav_sc0000102.1_g100.1. br* and *Pav_sc0002692.1_g030.1.mk*) of *PavBRI1-like* were notably induced, whereas another transcript, *Pav_sc0000617.1_g060.1.mk*, was inhibited in all three treatments. Notably, the transcript level of *PavBRI1* (*Pav_sc0000102.1_g170.1.br*) was markedly decreased in T treatment, but was markedly increased in S2 treatment.

#### 3.5.2 Gene expression profiles related to GA pathway

**Figure 6:**
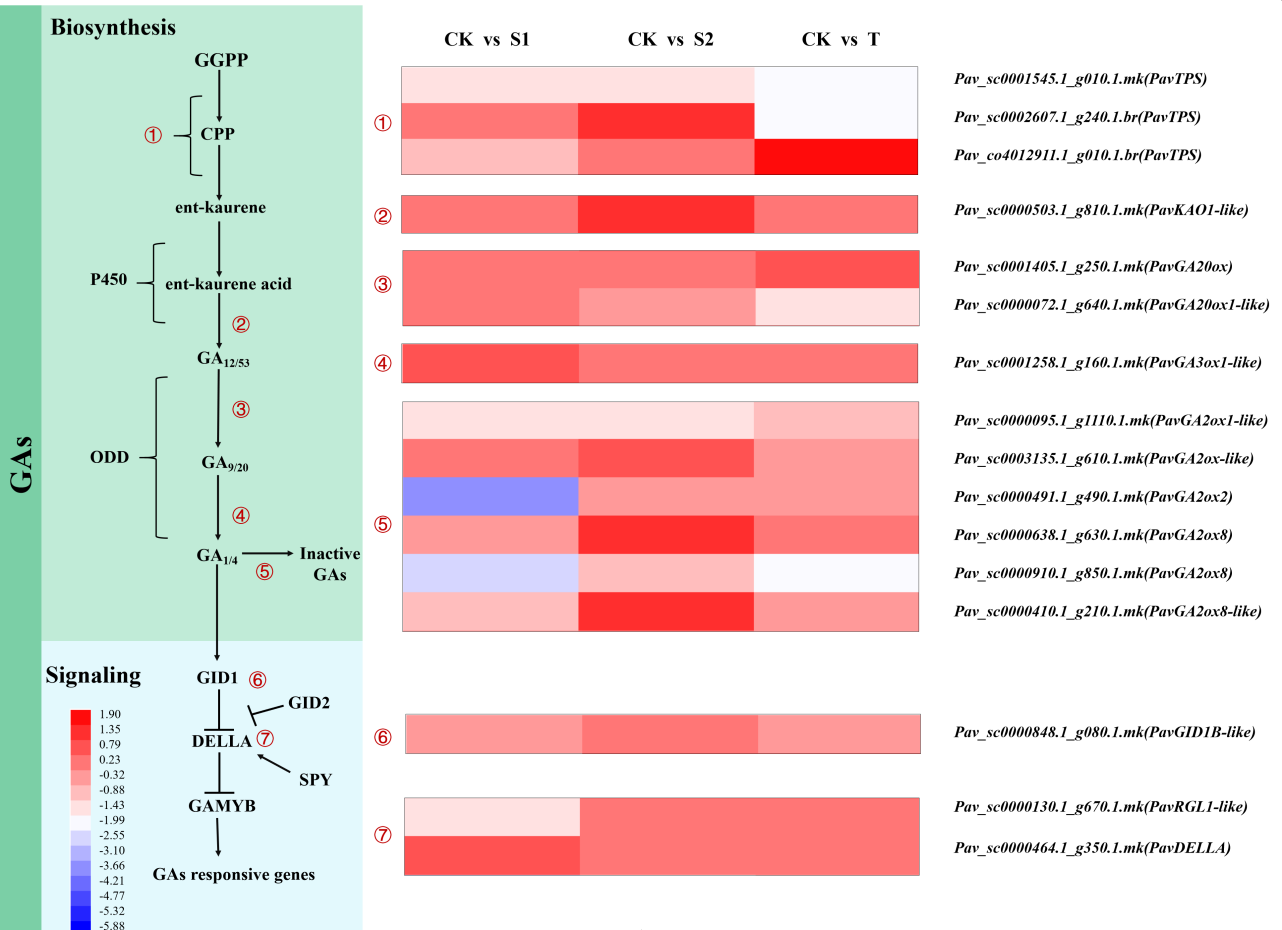
Heatmap represents expression profiles of DEGs related to gibberellin (GA) biosynthesis, deactivation, and signaling pathways. The scale of color intensity is shown in the lower-left quarter of the heatmap, representing the log_2_fold-change values. Fold-change refers to the ratio of gene expression levels in cherry rootstocks stems between control (CK) and treatment (S1/S2/T).

The expression levels of the DEGs involved in GA biosynthesis and signal transduction are shown in **Table S11** and **Figure 6**. We found that the expression patterns of terpene synthase (*PavTPS*), ent-kaurenoic acid oxidase 1-like (*PavKAO1-like*), gibberellin 20 oxidase (*PavGA20ox*), gibberellin 3-beta hydroxylase (*PavGA3ox*), and gibberellin 2-beta-dioxygenase (*PavGA2ox*) differed among three treatments and control groups. Some transcripts of *PavTPS* involved in the initial steps of GA biosynthesis exhibited different expression patterns under rac-GR24 and TIS108 treatments. The transcript *Pav_sc0001545.1_g010.1.mk* of *PavTPS* was markedly inhibited under all three treatments. The expression level of *PavTPS* (*Pav_sc0002607.1_g240.1.br*) was downregulated in T treatment but upregulated in S2 treatment. However, in another transcript, *Pav_co4012911.1_g010.1.br* of *PavTPS* was activated by T treatment. The transcript abundances of *PavKAO1-like* (*Pav_sc0000503.1_g810.1.mk*), *PavGA20ox8* (*Pav_sc0000638.1_g630.1.mk*), and *PavGA20ox8-like* (*Pav_sc000410.1_g210.1.mk*) were markedly increased in S2 treatment. The transcript abundance of *PavGA2ox1-like* (*Pav_sc0000095.1_g1110.1.mk*) was downregulated in S2 treatment, whereas that of *PavGA2ox-like* (*Pav_sc0003135.1_g610.1.mk*) was noticeably increased under the same treatment. The expression level of *PavGA2ox2* (*Pav_sc0000491.1_g490.1.mk*) was considerably downregulated in S1 treatment. The transcript abundance of *PavGA2ox8* (*Pav_sc0000910.1_g850.1.mk*) was markedly downregulated in S1 and T treatments, but no obvious change was observed in S2 treatment. The expression trend of this transcript was opposite to that of the GA_3_ content and stem length in S1 and T treatments. Thus, we speculate that SL may reduce the degradation of gibberellin by inhibiting the expression of *PavGA2ox8* (*Pav_sc0000910.1_g850.1.mk*) and then accumulate more GAs, thereby modulating stem growth.

#### 3.5.3 Gene expression profiles related to IAA pathway

**Figure 7:**
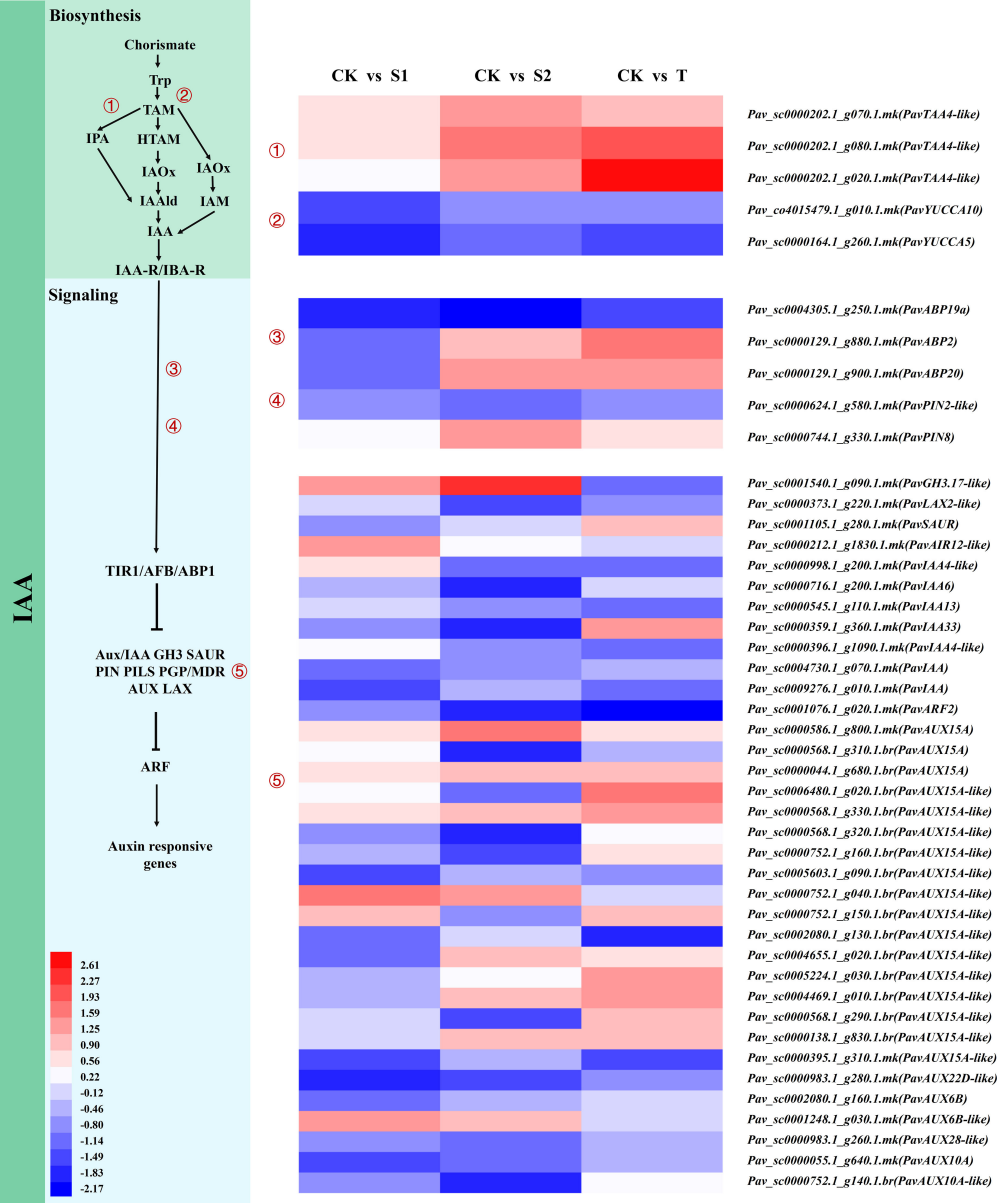
Expression profiles of differentially expressed genes (DEGs) related to 3-Indoleacetic acid (IAA) biosynthesis, and signaling pathways are shown by a heatmap. The scale of color intensity is shown in the lower-left quarter of the heatmap representing the log_2_fold-change values. Fold-change refers to the ratio of gene expression levels in cherry rootstocks stems between control (CK) and treatment (S1/S2/T).

IAA, the most important auxin in higher plants, plays a critical role in plant growth and development. We found that the transcript abundances of *PavTAA4-like* (*Pav_sc0000202.1_g070.1.mk, Pav_sc0000202.1_g080.1.mk*, and *Pav_sc0000202.1_g020.1.mk*) involved in IAA biosynthesis were significantly upregulated in S2 and T treatments (**Figure 7** and **Table S12**). *PavABP19a* (*Pav_sc0004305.1_g250.1.mk*) was notably inhibited by these treatments. *PavABP2* (*Pav_sc0000129.1_g880.1.mk*) and *PavABP20* (*Pav_sc0000129.1_g900.1.mk*) were suppressed in S1 treatment but were activated in T treatment. Notably, the auxin efflux transporter *PavPIN8* responded differently to three treatments. The transcript abundance of *PavPIN8* (*Pav_sc0000744.1_g330.1.mk*) was markedly upregulated in S2 treatment but did not exhibit obvious differences in S1 and T treatments. Similarly, the change in IAA content was accompanied by a similar tendency. *PavGH3.17-like* (*Pav_sc0001540.1_g090.1.mk*), which was involved in IAA biosynthesis, was activated by S1 and S2 treatments but suppressed by T treatment. The transcript abundances of several genes related to the auxin signaling pathway, including the auxin response protein *PavIAA13* (*Pav_sc0000545.1_g110.1.mk*), *PavIAA* (*Pav_sc0004730.1_g070.1.mk*), and auxin response factor 2 *PavARF2* (*Pav_sc0001076.1_g020.1.mk*), *PavAUX15A-like* (*Pav_sc0000395.1_g310.1.mk*), and *PavAUX22D-like* (*Pav_sc0000983.1_g280.1.mk*) were suppressed by all treatments. These results indicate that SLs affect the expression levels of IAA biosynthesis- and signaling pathway-related genes, implying that the interaction between SLs and IAA participates in the regulation of stem growth and development of cherry rootstocks.

#### 3.5.4 Gene expression profiles related to CTK pathway

Nine DEGs involved in the CTK biosynthesis and signal transduction pathways were selected for subsequent analysis (**Figure S10** and **Table S13**), including CTK synthase genes *LOGs* (*PavLOG5* and *PavLOG7*), CTK transporter genes (*PavPUP9*), CTK metabolism genes (*PavCKX1* and *PavCKX5*), ABC transporter G family members (*PavABCG23* and *PavABCG11-like*), and CTK signaling gene *ARRs* (*PavARR17, PavARR8*, *PavARR7*, and *PavARR5*). *PavLOG7* (*Pav_sc0000667.1_g210.1.mk*) was considerably inhibited by S2 treatment, whereas *PavLOG5* (*Pav_sc0000244.1_g050.mk*) was notably activated by S1 and S2 treatments. In this study, *PavCKX1* (*Pav_sc0000096.1_g120.1.mk*) was markedly induced by S2 treatment, whereas *PavCKX5* (*Pav_sc0002208.1_g430.1.mk*) was remarkably activated by T treatment. The expression level of *PavPUP9* (*Pav_sc0000108.1_g1020.1.mk*) was significantly upregulated in S2 treatment. The transcript abundances of *PavABCG23* (*Pav_sc0007696.1_g040.1.mk*), *PavABCG11-like* (*Pav_sc0006296.1_g030.1.mk*), and *PavAHP4-like* (*Pav_sc0000800.1_g510.1.mk*) were notably upregulated in S2 and T treatments but did not show a significant difference in S1 treatment. The expression levels of *PavABCG15-like* (*Pav_sc0001391.1_g020.1.br*), *PavAHP6-like* (*Pav_co4027403.1_g010.1.mk*), and *PavARR5* (*Pav_sc0000138.1_g610.1.mk*) were markedly increased in three treatment groups.

#### 3.5.5 Gene expression profiles related to ABA pathway

**Figure 8:**
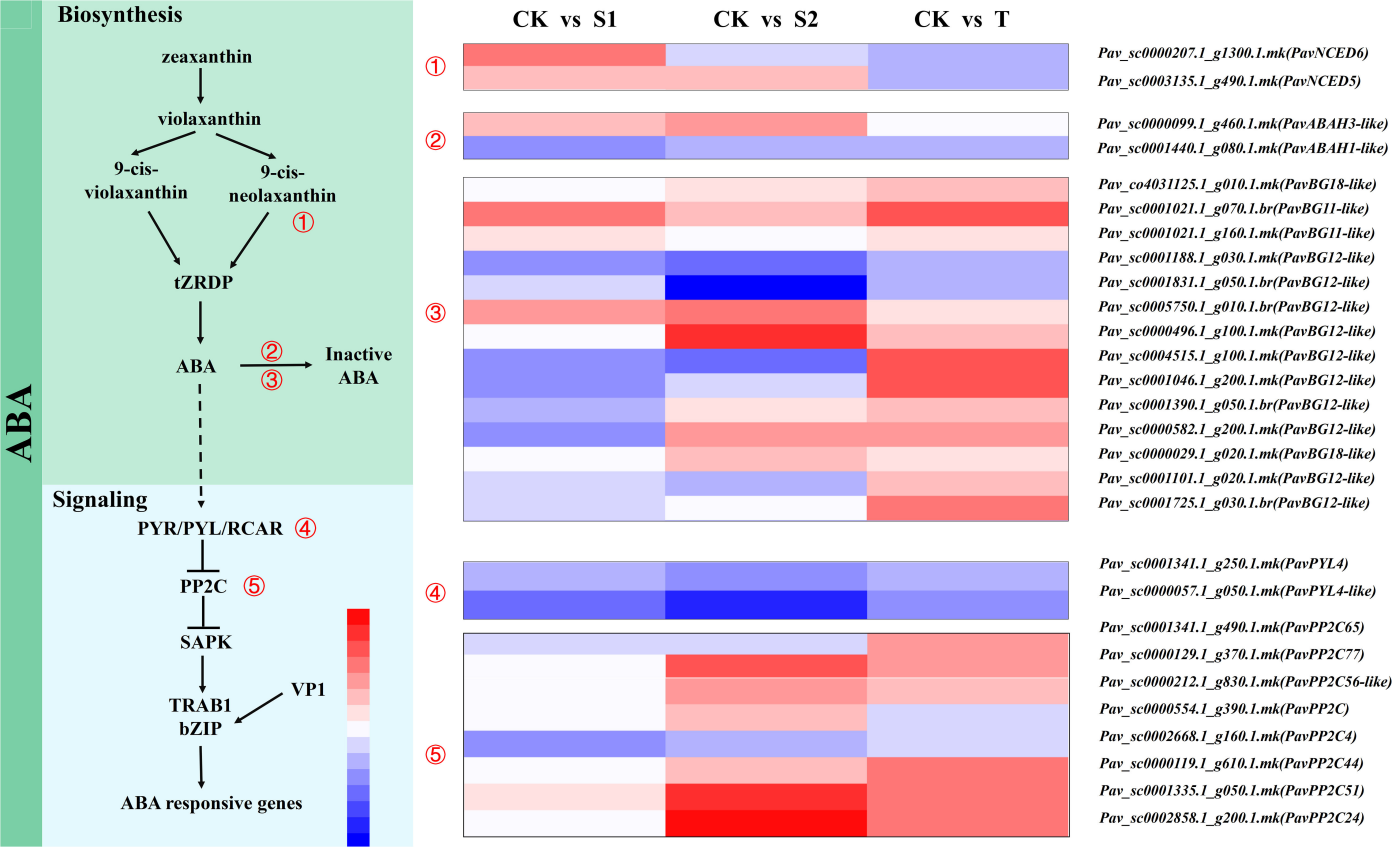
Expression profiles of differentially expressed genes (DEGs) related to abscisic acid (ABA) biosynthesis and signaling pathways are represented by a heatmap. The scale of color intensity is shown in the lower-left quarter of the heatmap representing the log_2_fold-change values. Fold-change refers to the ratio of gene expression levels in cherry rootstocks stems between control (CK) and treatment (S1/S2/T).

We analyzed the transcript abundances of some DEGs involved in the ABA pathways (**Figure 8** and **Table S14**). ABA synthase genes *NCED* (*PavNCED5* and *PavNCED6*), beta-glucosidase (*PavBG11-like* and *PavBG12-like*), ABA signaling genes *PP2Cs* (*PavPP2C4*, *PavPP2C44*, *PavPP2C51*, *PavPP2C56-like*, *PavPP2C65*, and *PavPP2C77*) were used as representative genes. The expression level of *PavNCED6* (*Pav_sc0000207.1_g1300.1.mk*) was significantly upregulated only in S1 treatment. However, the transcript abundance of *PavNCED5* (*Pav_sc0003135.1_g490.1.mk*) was remarkably upregulated in both S1 and S2 treatments. *PavBG11-like* (*Pav_sc0001021.1_g070.1.br*) and *PavBG12-like* (*Pav_sc0005750.1_g010.1.br*) were markedly activated in all three treatment groups. Additionally, two transcripts (*Pav_sc0001188.1_g030.1.mk *and *Pav_sc0001831.1_g050.1.mk*) of *PavBG12-like* were markedly inhibited by S2 treatment. The expression levels of *PavABAH1-like* (*Pav_sc0001440.1_g080.1.mk*) and *PavPP2C4* (*Pav_sc0002668.1_g160.1.mk*) were notably down regulated by S1 treatment.

### 3.6 DEGs related to stem growth or cell cycle

**Figure 9:**
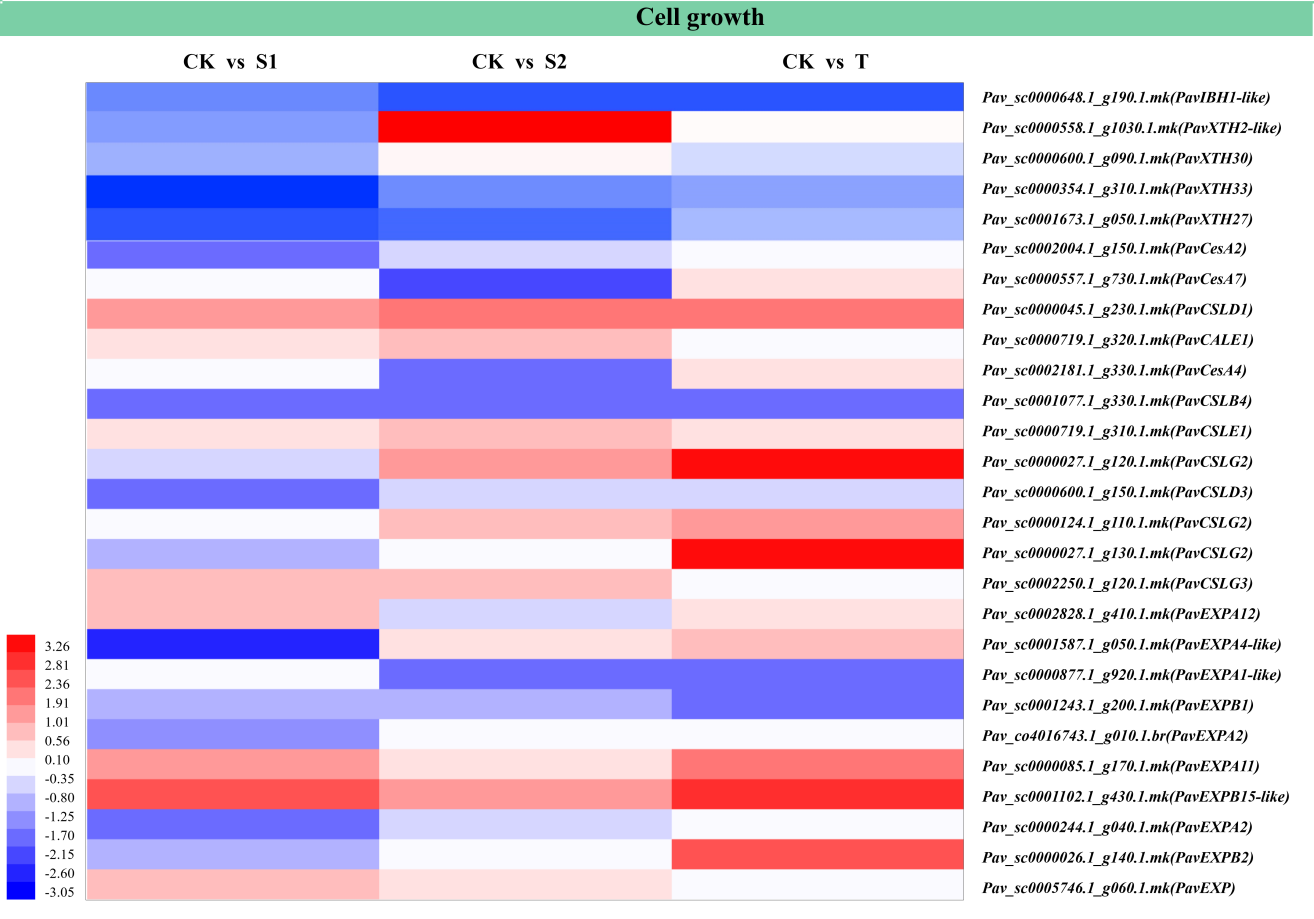
Heatmap showed the expression profiles of differentially expressed genes (DEGs) related to cell growth and cell expansion. The scale of color intensity is shown in the lower-left corner of the heatmap representing the log_2_fold-change values. Fold-change refers to the ratio of gene expression levels in cherry rootstocks stems between control (CK) and treatment (S1/S2/T).

The results of the analysis of six stem development-related DEGs are shown in **Figure 9** and **Table S15**. These DEGs included transcription factor IBH1-like 1 (*PavIBH1-like*), cellulose synthase-like protein (*PavCSL*), and expansin-like (*PavEXP*). The transcript abundances of *PavIBH1-like* (*Pav_sc0000648.1_g190.1.mk*) and *PavEXPA1-like* (*Pav_sc0000877.1_g920.1.mk*) were markedly decreased in S2 and T treatments. In contrast, the transcript abundances of *PavXTH2-like* (*Pav_sc0000558.1_g1030.1.mk*), *PavCSLG2* (*Pav_sc0000027.1_g120.1.mk*), and *PavCSLG2* (*Pav_sc0000124.1_g110.1.mk*) were significantly increased in S2 and T treatments. The transcript abundances of *PavEXPA12* (*Pav_sc0002828.1_g410.1.mk*) and *PavEXP* (*Pav_sc0005746.1_g060.1.mk*) were markedly upregulated under S1 treatment, whereas the transcript abundances of *PavXTH33* (*Pav_sc0000354.1_g310.1.mk*), *PavXTH27* (*Pav_sc0001673.1_g050.1.mk*), *PavCesA2* (*Pav_sc0002004.1_g150.1.mk*), *PavCSLD3* (*Pav_sc0000600.1_g150.1.mk*), *PavEXPA2* (*Pav_co4016743.1_g010.1.br*), and *PavEXPA2* (*Pav_sc0000244.1_g040.1.mk*) were markedly downregulated under S1 treatment. The transcript abundances of *PavXTH30* (*Pav_sc0000600.1_g090.1.mk*) and *PavCSLE1* (*Pav_sc0000719.1_g310.1.mk*) were markedly upregulated, whereas the transcript abundances of *PavCesA7* (*Pav_sc0000557.1_g730.1.mk*) and *PavCesA4* (*Pav_sc0002181.1_g330.1.mk*) were markedly downregulated under S2 treatment. The transcript abundances of *PavSOC1-like* (*Pav_sc0001405.1_g300.1.mk*), *PavCSLG2* (*Pav_sc0000027.1_g130.1.mk*), and *PavEXPB2* (*Pav_sc0000026.1_g140.1.mk*) were markedly upregulated, whereas that of *PavEXPB1* (*Pav_sc0001243.1_g200.1.mk*) was markedly downregulated under T treatment. *PavCSLD1* (*Pav_sc0000045.1_g230.1.mk*) and *PavEXPB15-like* (*Pav_sc0001102.1_g430.1.mk*) were notably activated, whereas *PavCSLB4* (*Pav_sc0001077.1_g330.1.mk*) was markedly inhibited by all three treatments.

### 3.7 Validation of DEGs using quantitative reverse transcription PCR

**Figure 10:**
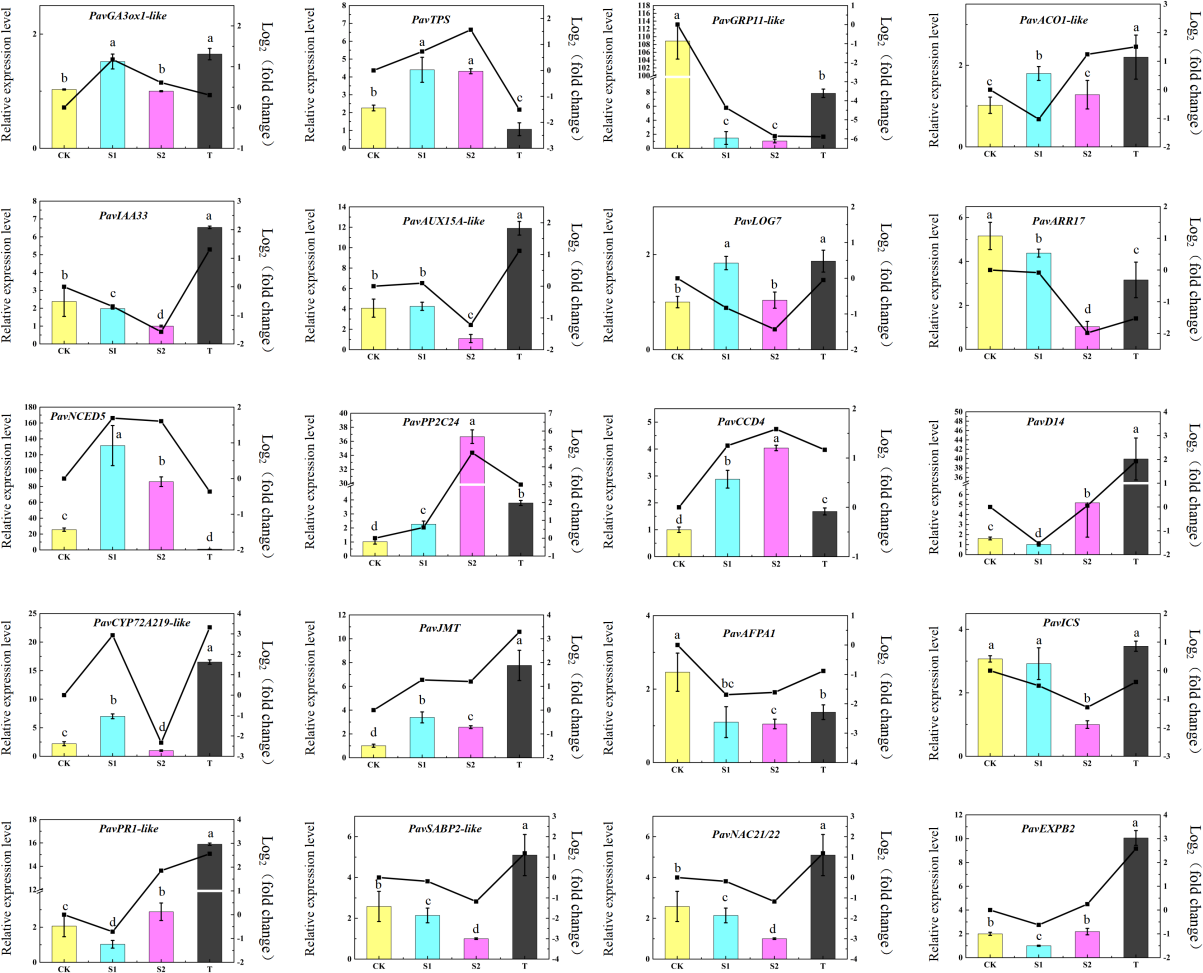
Quantitative real-time polymerase chain reaction (qRT-PCR) validation of gene expression levels of 20 selected DEGs related to different hormone pathways, cell elongation, and cell development. The data represent means ± standard deviation (SD) of three replicates. Statistical significance was determined by one-way ANOVA; significant differences among means (LSD, *p* < 0.05) are indicated by different lowercase letters.

In this study, 20 DEGs were screened for qRT-PCR assay to verify the repeatability and accuracy of the RNA-seq data in the cherry rootstocks. The expression trends observed in the qRT-PCR assay are similar to those observed in the transcriptome data (**Figure 10**). The results showed that the mRNA levels of three DEGs, *PavGRP11-like* (*Pav_sc0000848.1_g820.1.mk*), *PavARR17* (*Pav_sc0001582.1_g370.1.mk*), and *PavAFPA1* (*Pav_sc0000358.1_g280.1.mk*) were the highest in the CK group. In contrast, five DEGs*, PavACO1-like* (*Pav_sc0000027.1_g360.1.br*), *PavPP2C24* (*Pav_sc0002858.1_g200.1.mk*), *PavCCD4* (*Pav_sc0000354.1_g230.1.mk*), and *PavJMT* (*Pav_sc0002208.1_g770.1.mk*) were induced in all three treatments. The transcript abundances of *PavD14* (*Pav_sc0000127.1_g100.1.mk*), *PavEXPB2* (*Pav_sc0000026.1_g140.1.mk*), and *PavPR1* (*Pav_sc0000568.1_g700.1.br*) were lowest under S1 treatment. The expression levels of the three genes also exhibited similar expression trends. *PavNCED5* (*Pav_sc0003135.1_g490.1.mk*) and *PavTPS* (*Pav_sc0002607.1_g240.1.br*) were markedly activated in S1 and S2 treatments, whereas they were markedly inhibited in T treatment. Conversely, *PavIAA33* (*Pav_sc0000359.1_g360.1.mk*) and *PavSABP2-like* (*Pav_sc0000348.1_g320.1.mk*) were inhibited in S1 and S2 treatments but were notably activated in T treatment. Collectively, the qRT-PCR results showed that the expression levels of cell growth- and expansion-related genes were substantially affected by treatment with TIS108 or rac-GR24.

### 3.8 Correlation between endogenous hormone content and transcript level

**Figure 11:**
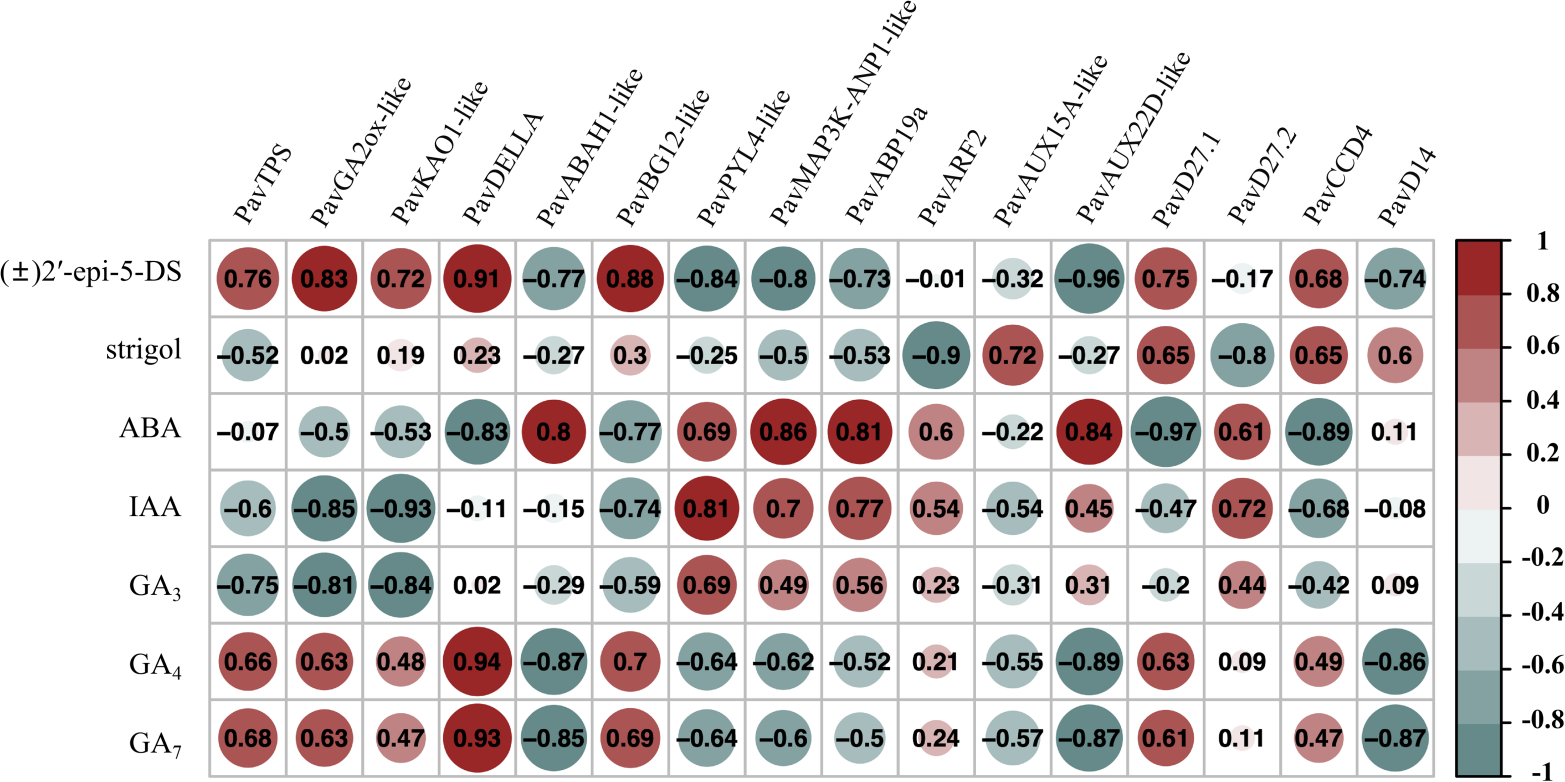
Correlation analysis between endogenous hormone contents and gene expression levels (*p* < 0.05).

To analyze the relationship between gene expression levels and phytohormone content, the corrplot package was used to analyze the correlation between phytohormone levels and transcript abundance (**Figure 11**, **Figure S10** and **Table S16**). The transcript abundances of *PavKAO1*-*like* (*Pav_sc0000503.1_g810.1.mk*), *PavDELLA* (*Pav_sc0000464.1_g350.1.mk*), *PavARF2* (*Pav_sc0001076.1_g020.1.mk*), *PavAUX22D-like* (*Pav_sc0000983.1_g280.1.mk*), and *PavD27.1* (*Pav_sc0000589.1_g220.1.mk*) from the random selected 16 transcripts were closely related to six phytohormone contents, with correlation coefficients approaching 0.90 (**Figure 11**). The transcript abundance of *PavKAO1-like* (*Pav_sc0000503.1_g810.1.mk*) was negatively correlated with the endogenous IAA content (r = −0.93, *p* < 0.05). The transcript abundance of *PavDELLA* (*Pav_sc0000464.1_g350.1.mk*) was significantly positively correlated with endogenous [(±)2-epi-5-DS], GA_4_, and GA_7_ content (r = 0.91, r = 0.94, r = 0.93, *p* < 0.05, respectively; **Figure 11**). The transcript abundance of *PavARF2* (*Pav_sc0001076.1_g020.1.mk*) was negatively correlated with endogenous strigol content (r = −0.90, *p* < 0.05). The transcript abundance of *PavAUX22D-like* (*Pav_sc0000983.1_g280.1.mk*) was negatively correlated with the endogenous (±)2’-epi-5-DS content (r = −0.96, *p* < 0.05). The transcript abundance of *PavD27.1* (*Pav_sc0000589.1_g220.1.mk*) was negatively correlated with the endogenous ABA content (r = −0.97, *p* < 0.05). Moreover, The transcript abundance of *PavCCD4* (*Pav_sc0000354.1_g230.1.mk*) was negatively correlated with the endogenous ABA content (r = −0.89, *p* < 0.05; **Figure 11**). The correlation between phytohormone content and gene expression levels requires verification through additional methods, such as proteomics and metabolomics.

## 4 Discussion

### 4.1 Aboveground morphological traits of cherry rootstocks in response to SL-analog and SL biosynthetic inhibitor

SLs plays an important role in controlling many aspects of plant development, including seedling development, branch branching and leaf senescence (Liu et al., 2015; Yang et al., 2020). At 30 DAT, the stem length of plantlets following 0.1 μM rac-GR24 (S1) or 10 μM TIS108 (T) treatment was markedly higher than that of the control group (**Figure 1**). We observed that the length of the third and fourth internodes after rac-GR24 or TIS108 treatment was significantly higher than that of the control group (**Figures S1A–C**). Moreover, the results of stem slices at 30 DAT also support that SL inhibitor may promote cell size (**Figures S1D–F**). Similarly, GA_3_ levels in stems increased considerably after S1 or T treatment compared with the control group. GAs are essential phytohormones that participate in the regulation of plant stem elongation (Raskin and Kende, 1984; Kende et al., 1998; Yin et al., 2007). Its deficiencies in GA signaling or biosynthesis result in dwarfism in plants (Sakamoto et al., 2004; Ueguchi-Tanaka et al., 2005). Thus, it is likely that SLs modulate endogenous GA_3_ levels to regulate stem elongation in cherry rootstocks. A previous report showed that SLs accelerate the accumulation of bioactive GAs, including GA_4_ and GA_1_ (Toh et al., 2012); however, they do not influence GA signaling or metabolism in Pea (De Saint Germain et al., 2013). We found that high and low concentrations of rac-GR24 promoted the accumulation of bioactive GA_4_, which is consistent with the previous report (Toh et al., 2012). Previous studies have shown that SLs increased the stem diameter of plants (*Arabidopsis thaliana*, maize, and so on) (Agusti et al., 2011; Guan et al., 2012; Screpanti et al., 2016). However, our results showed that rac-GR24 effectively reduced the stem diameter, which is inconsistent with previous reports. This may because they belong to different species, and have different physiological, genetic, and developmental differences. Exogenous 0.1 μM GR24 treatment improved the total chlorophyll content of rape seedlings under low-temperature conditions (Zhang et al., 2020). However, the total chlorophyll content of Krymsk^®^5 treated with 0.1 μM rac-GR24 did not exhibit significant differences compared with the control group in the present study. We speculated that this divergence may have resulted from the difference in the testing temperature conditions.

### 4.2 Regulation of stem growth and development by hormone signaling and metabolism

Phytohormone play critical roles in the regulation of plant growth and development (Wani et al., 2016). Our findings show that a number of DEGs are involved in phytohormone signaling and metabolism pathways. SLs are crucial in regulating plant growth and development (Marzec and Muszynska, 2015) as they enhance plant adaptability to drought, low phosphorus and nitrogen levels, and other stresses, which can promote yield improvement (Mostofa et al., 2018; Duan et al., 2019; Wang et al., 2020). Among the various phytohormones, auxin affects stem elongation and regulates meristem formation and fate, and has been proven to be an important hormone that restricts plant architecture (Gallavotti, 2013). Previous reports showed that exogenous rac-GR24 reduces endogenous IAA synthesis by inhibiting the expression level of *YUCCA* (Zhuo et al., 2021). In our study, the application of exogenous rac-GR24 decreased the IAA content of the stem. The decrease in IAA content of the stem was noticeably higher with a higher concentration of rac-GR24, which is consistent with the findings of Zhuo et al. (2021). Auxin homeostasis is dominated by the putative auxin efflux carrier component 8 (*PavPIN8*), which is involved in stem and hypocotyl development in *A. thaliana.* Our results show that *PavPIN8* (*Pav_sc0000744.1_g330.1.mk*) was substantially induced by treatment with 10 μM rac-GR24, which displayed a similar tendency to the change in IAA content. In conclusion, we speculated that rac-GR24 affected IAA content in cherry rootstocks stems by regulating the transcriptional of *PavPIN8*.

Recent reports have demonstrated that exogenous rac-GR24 inhibits the expression of the CTK synthesis-related gene *LOG1* (Cui, 2015) and induces the degradation of CTK oxidases to reduce the content of CTK (Duan et al., 2019). In this study, after exogenous rac-GR24 treatment, we found that the stem diameter significantly decreased. However, after S2 treatment, no significant differences were observed. At 30 DAT, the cytokinin synthesis-related genes *PavLOG7* (*Pav_sc0000667.1_g210.1.mk*) and *PavIPT-like* (*Pav_sc0001051.1_g170.1.mk*) decreased significantly under rac-GR24 treatment, whereas exogenous TIS108 only had a marginal effect on the expression levels of the two transcripts. This suggests that exogenous rac-GR24 inhibited lateral growth of the stem by inhibiting the expression of cytokinin synthesis genes *PavLOG7* and *PavIPT-like*, thereby reducing stem diameter. Additionally, a previous report showed that the expression level of *OsIPT* gene was not significantly induced by GR24 treatment in rice (Xu et al., 2015), which is inconsistent with our results. We speculate that this may be due to the different genetic backgrounds of rice and cherry rootstock Krymsk^®^5.

In addition to IAA, GA plays a crucial role in stem elongation (Yin et al., 2007; Gallavotti, 2013). Defects in GA biosynthesis and signal transduction can lead to rice dwarfing (Sakamoto et al., 2004; Ueguchi-Tanaka et al., 2005; Yin et al., 2007). The rice GA mutant is not sensitive to exogenous rac-GR24 treatment, unlike the wild type, indicating that SLs often crosstalk with GA signaling (Ito et al., 2017; Zou et al., 2019). GA_4_ levels are correlated with the final internode length of hybrid aspens (Israelsson et al., 2004). In the stems of cherry rootstocks treated with S1, we observed a notable increase in GA_4_ content. In the present study, 0.1 μM rac-GR24 promoted the transcription of *PavGA3ox1-like* (*Pav_sc0001258.1_g160.1.mk*), which was consistent with a previous report (Katyayini et al., 2020). Additionally, the S1 and T treatment significantly downregulated the expression of GA metabolism gene *PavGA2ox8* (*Pav_sc0000910.1_g850.1.mk*). Thus, we speculated that there is an crosstalk between SLs and GA involved in the regulation of stem elongation in the cherry rootstocks. However, the interaction between SLs and GAs has not been fully elucidated, and needs to be resolved by molecular biology and metabolomic methods in future studies.

### 4.3 Multiple hormrne pathways are regulated by cell growth related genes

SLs can enhance the elongation and cell wall thickness of cotton fiber cells, and then promote the length and strength of fiber cells (Tian Z et al., 2022). We want to explore whether SL can promote stem elongation by regulating cell growth. Cellulose synthase-like protein G is a Golgi-localized protein beta-glycan synthase, which forms the backbone of the non-cellulosic polysaccharide of plant cell wall polymerization (Little et al., 2019; Galinousky et al., 2020). In the current study, rac-GR24 treatment activated the transcription of *PavCSLG3* (*Pav_sc0002250.1_g120.1.mk*), which encodes the cell synthesis-like protein G3. Treatment with TIS108 promoted the transcription of *PavCSLG2* (*Pav_sc0000027.1_g130.1.mk*). Moreover, S2 and T treatments increased the expression levels of *PavCSLG2* (*Pav_sc0000027.1_g120.1.mk* and *Pav_sc0000124.1_g110.1.mk*). This indicates that SLs are likely to regulate the formation of cell walls by affecting the expression levels of *PavCSLG2* and *PavCSLG3*. *EXPA* and *EXPB* regulate the relaxation and elongation of cell walls in plants (Breen et al., 2010). Following S1 and T treatments, the expression levels of *PavEXPA11* (*Pav_sc0000085.1_g170.1.mk*) and *PavEXPB 15-like* (*Pav_sc0001102.1_g430.1.mk*) increased significantly, indicating that SLs might regulate stem elongation by affecting the relaxation and elongation of the cell wall. Previous studies have reported that *AtIBH1-like* negatively regulates cell elongation (Ikeda et al., 2012). Following treatments with 10 μM TIS108 and rac-GR24, in the current study, the expression of *PavIBH1-like* (*Pav_sc0000648.1_g190.1.mk*) decreased significantly, implying that SLs may regulate cell elongation by affecting the expression level of *PavIBH1-like*, thereby regulating stem growth. In conclusion, SLs may regulate the growth and development of stems by affecting the expression levels of cell growth genes.

## 5 Conclusions

In this study, stem length and diameter, Fw, and chlorophyll content exhibited varying degrees of change following TIS108 or rac-GR24 treatment, which enabled the alteration of endogenous phytohormone levels. The GA_3_ content of cherry rootstocks treated with S1 and T were significantly higher than that of the control group. In addition, the change trend of GA_3_ content in stems after these two treatments are highly similar to that of stem length of cherry rootstocks, which imply that the crosstalk between SLs and GA is involved in regulating stem elongation of cherry rootstocks. Stem paraffin sections also supported the characteristic phenotypic changes in the stem under rac-GR24 or TIS108 treatment. A comprehensive understanding of stem growth and development in response to rac-GR24 or TIS108 treatment was obtained by transcriptome sequencing. The results of RNA-seq revealed several DEGs, such as *CKX, LOG, YUCCA, AUX*, and *EXP*, which play vital roles in stem growth of cherry rootstocks. Our study demonstrated that SLs modulate stem growth and development of cherry rootstocks by interacting with other hormone signaling pathways. This study provides a theoretical basis for the use of SLs to regulate plant height, and optimization for cherry dwarfing and high-density cultivation.

## Data availability statement

The original contributions presented in the study are included in the article/**Supplementary Material**, further inquiries can be directed to the corresponding author/s.

## Author contributions

XL: Investigation, writing – review and editing, methodology. YX: Investigation and methodology. WS: Writing-review and editing, formal analysis. JW: methodology, software, and formal analysis. YG: methodology. LW: Methodology. WX: Data curation. SW: Conceptualization, resources, and supervision. SJ: Conceptualization, resources, supervision. CZ: Conceptualization, resources, supervision, project administration. All authors contributed to the article and approved the submitted version.
