# Supplementary figures and images for: Strigolactones modulate stem length and diameter of cherry rootstocks through interaction with other hormone signaling pathways
Source: Front Plant Sci. 2023 Feb 9;14:1092654. doi: 10.3389/fpls.2023.1092654 (PMC9948674; doi:10.3389/fpls.2023.1092654)

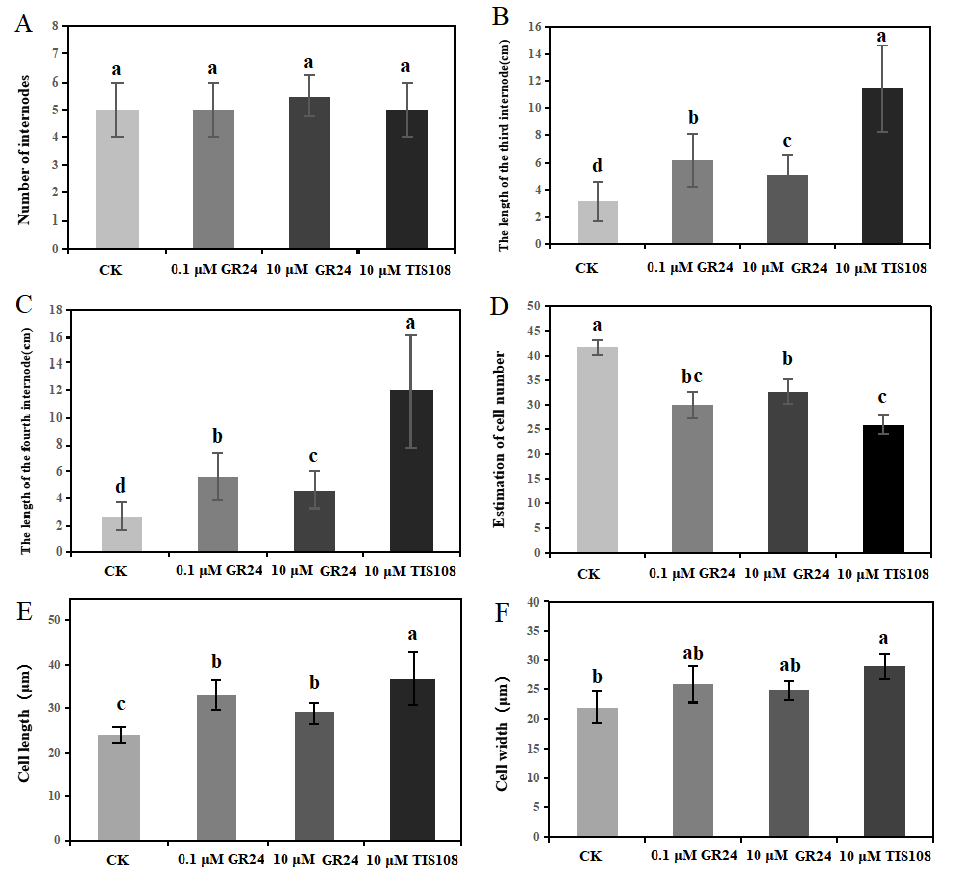

Supplement: Supplementary file 1 [file Image_1.tif]

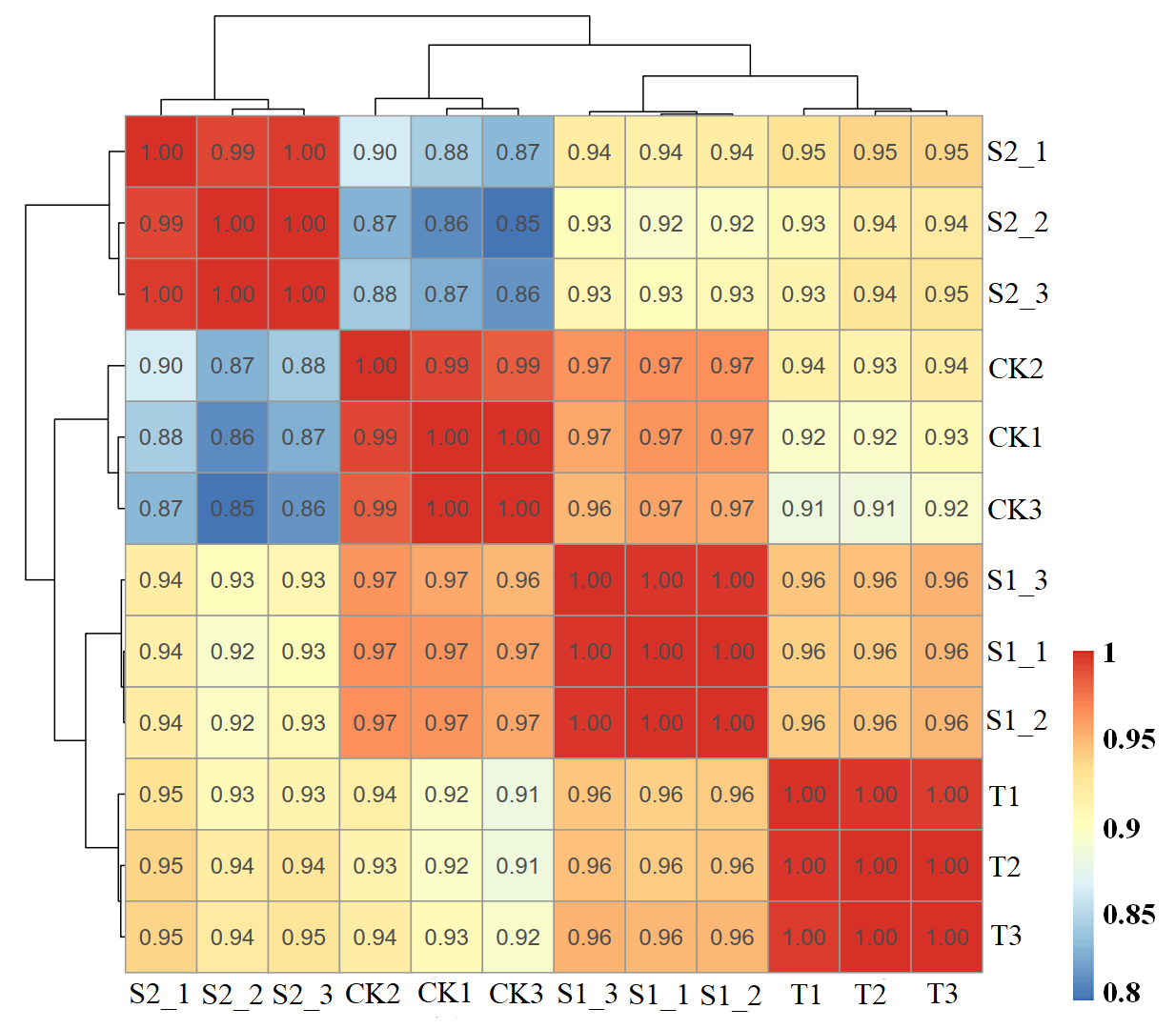

Supplement: Supplementary file 2 [file Image_2.tif]

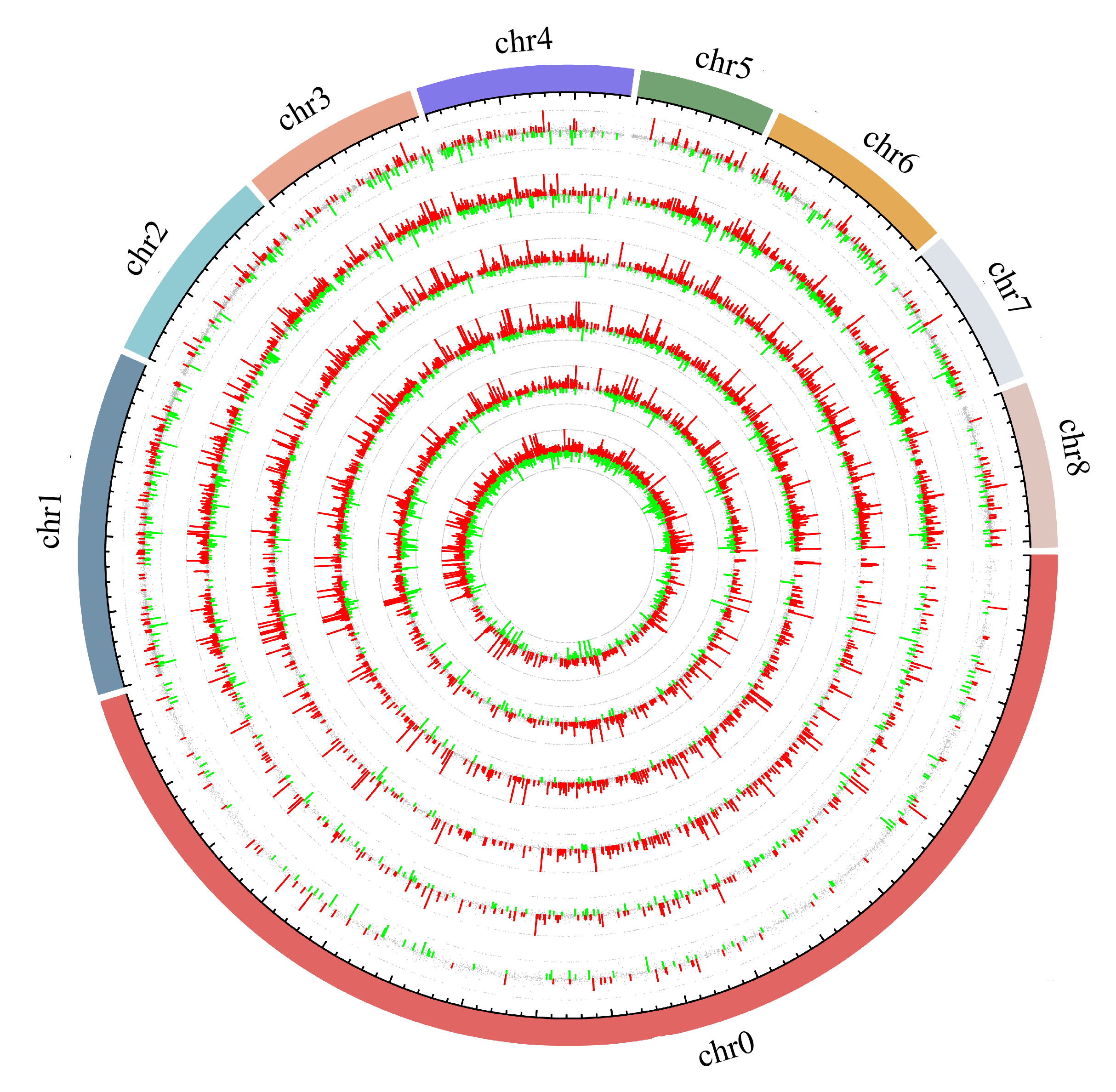

Supplement: Supplementary file 3 [file Image_3.tif]

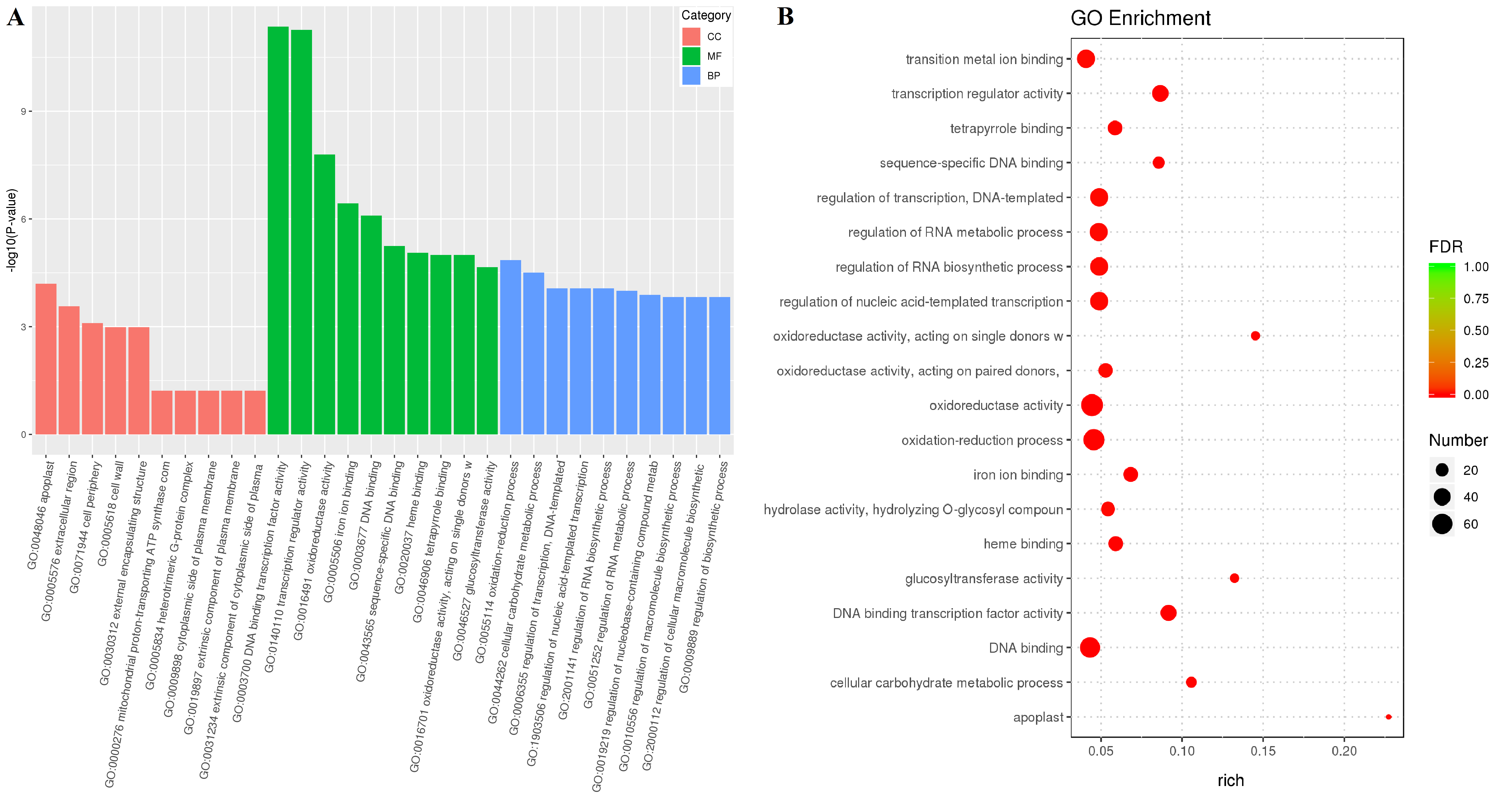

Supplement: Supplementary file 4 [file Image_4.tif]

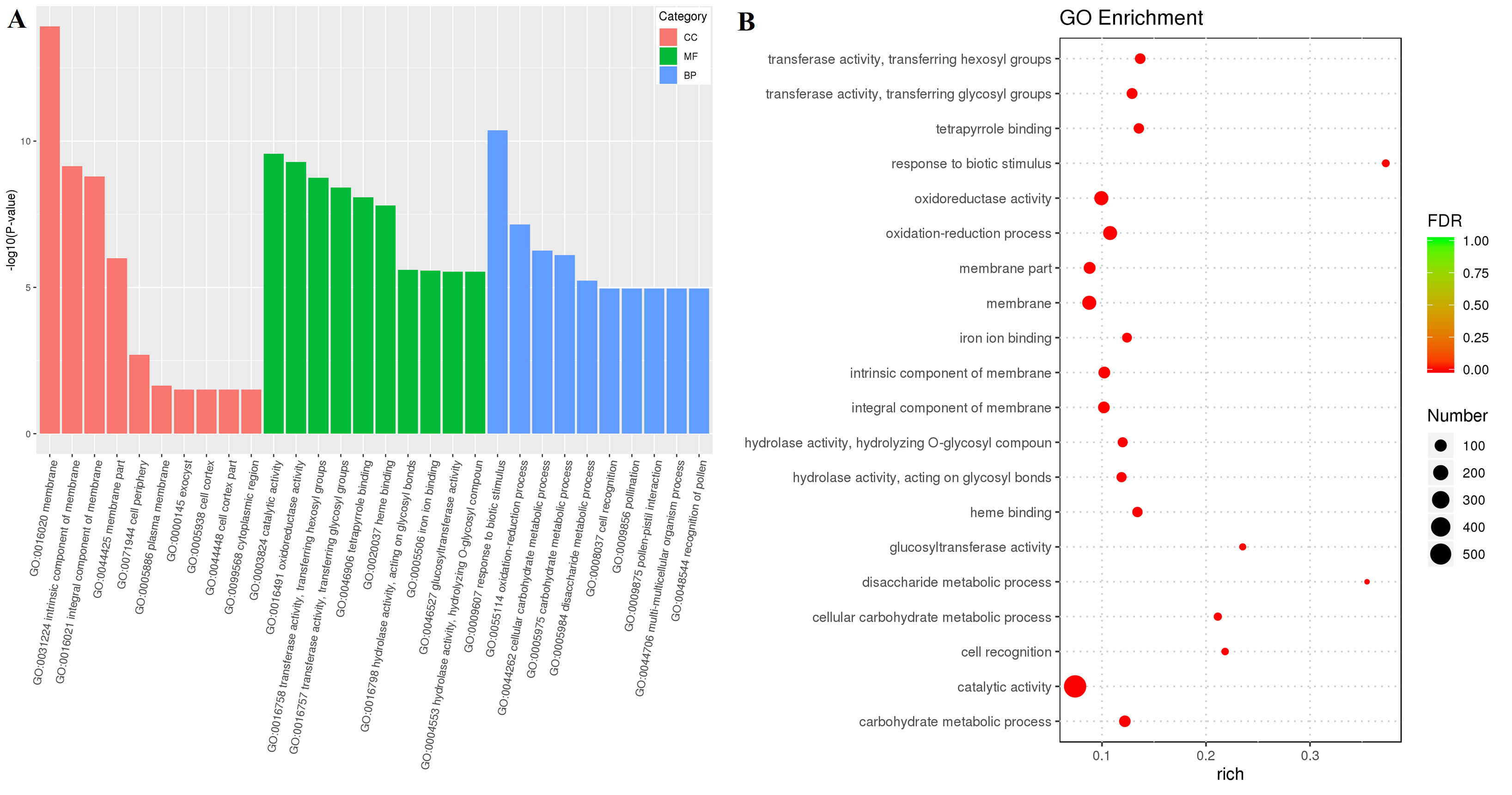

Supplement: Supplementary file 5 [file Image_5.tif]

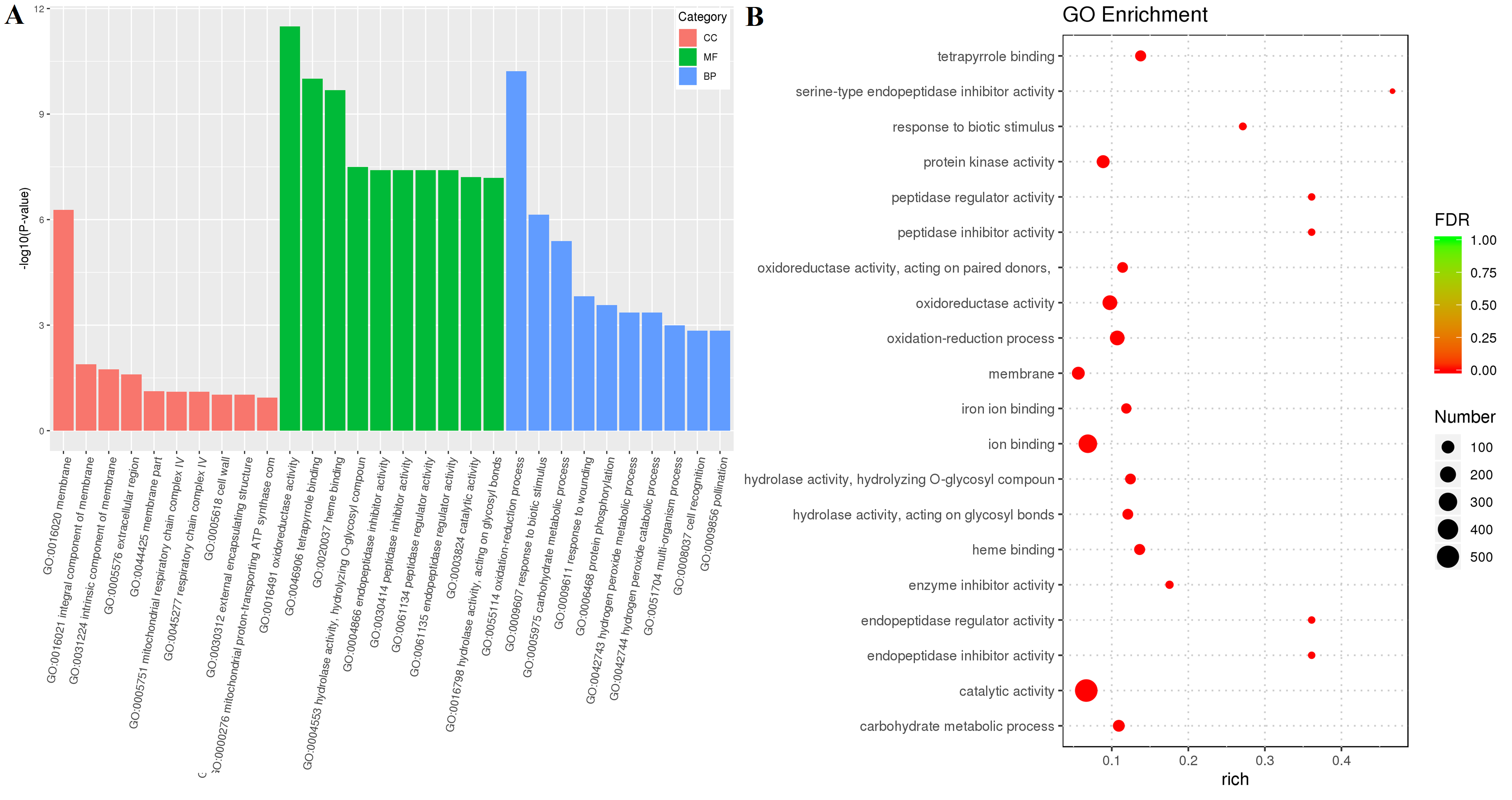

Supplement: Supplementary file 6 [file Image_6.tif]

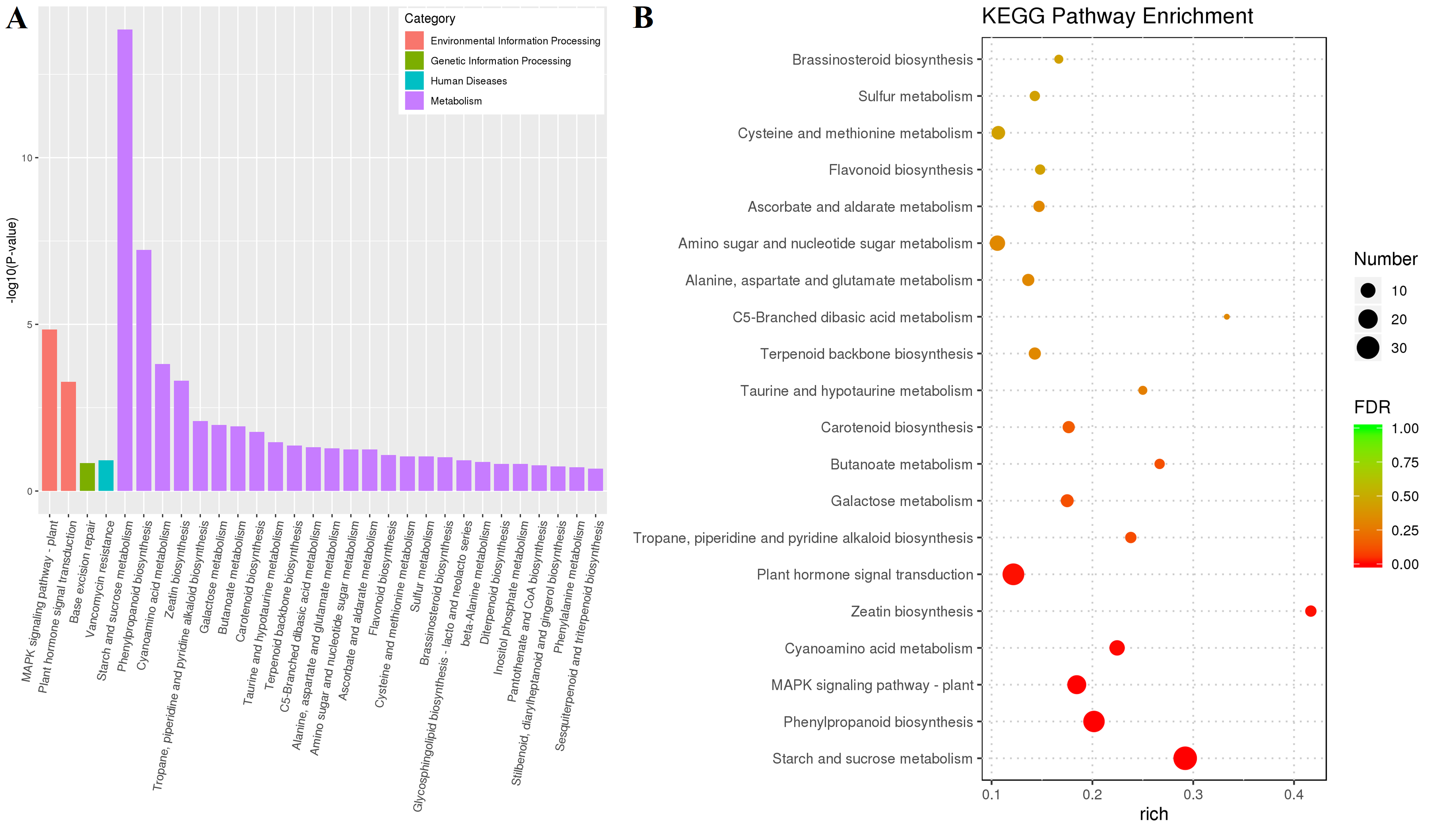

Supplement: Supplementary file 7 [file Image_7.tif]

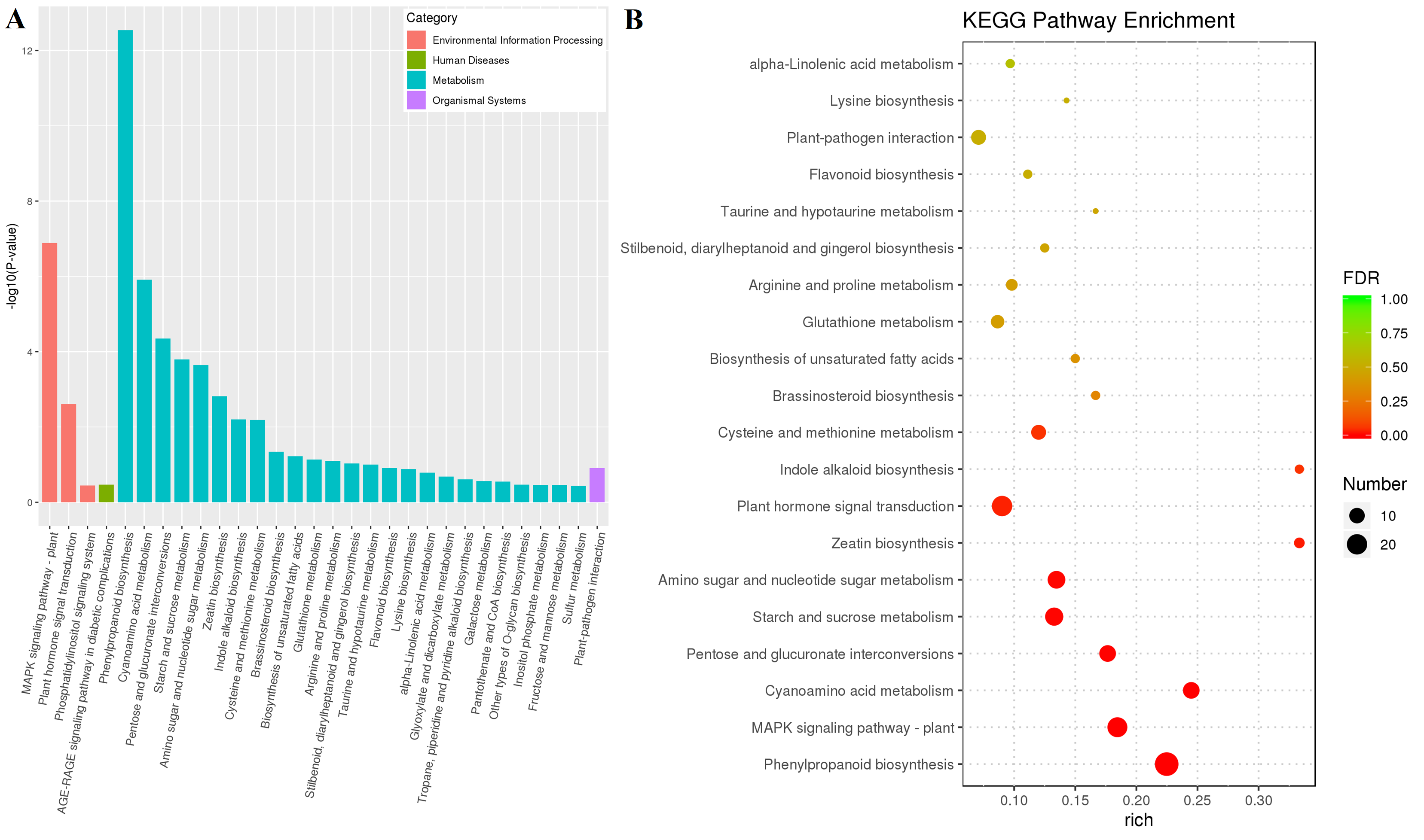

Supplement: Supplementary file 8 [file Image_8.tif]

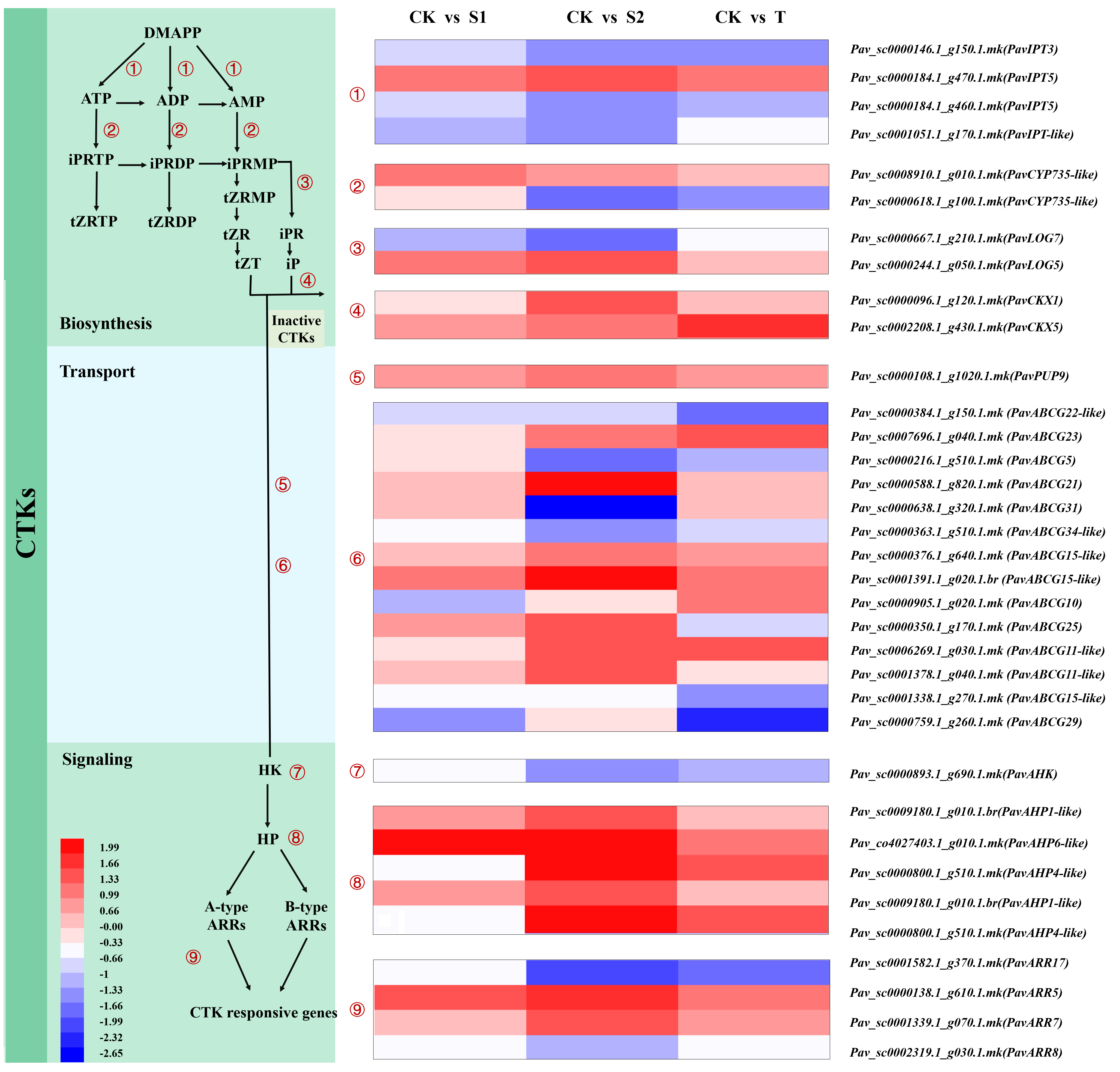

Supplement: Supplementary file 9 [file Image_9.tif]

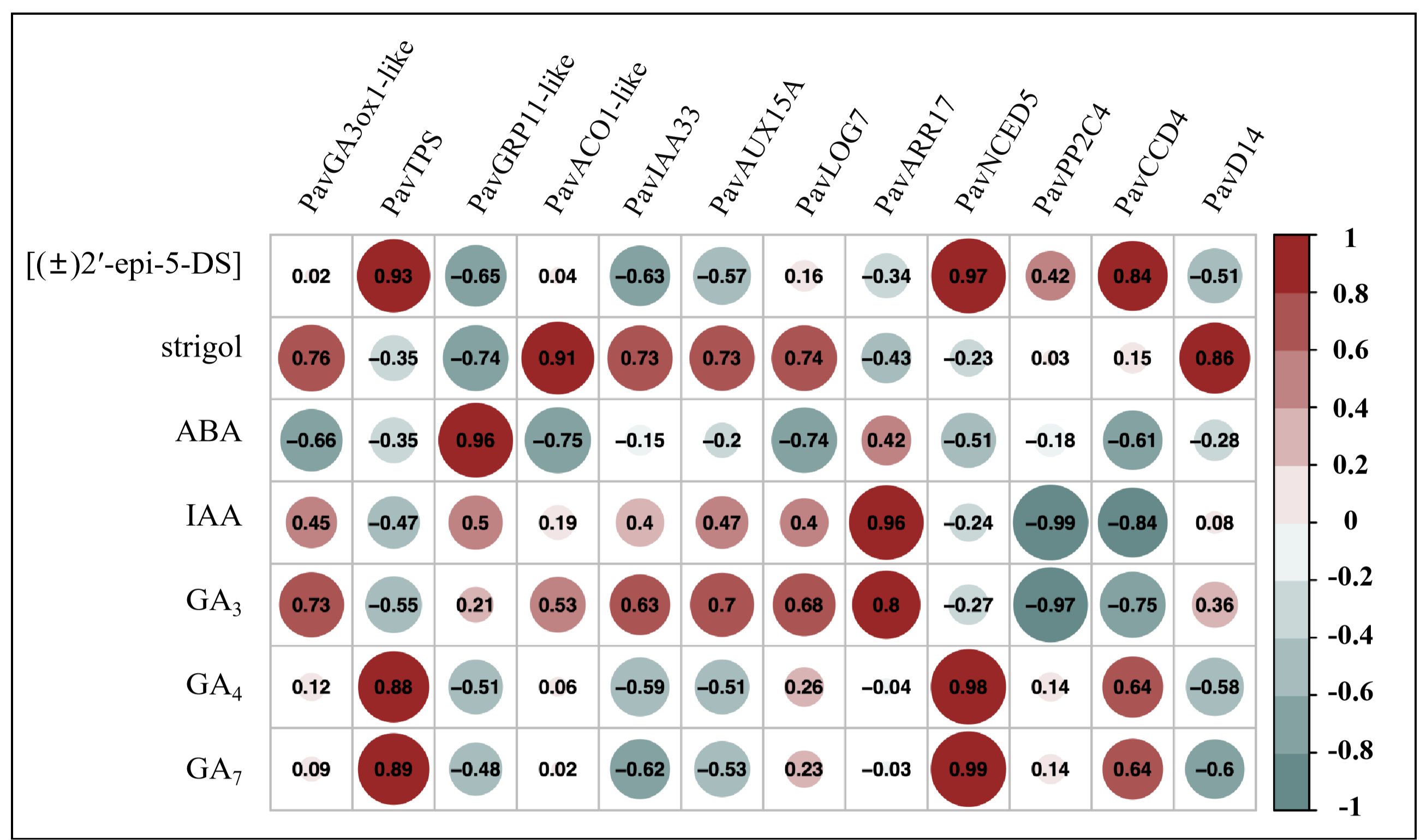

Supplement: Supplementary file 10 [file Image_10.tif]

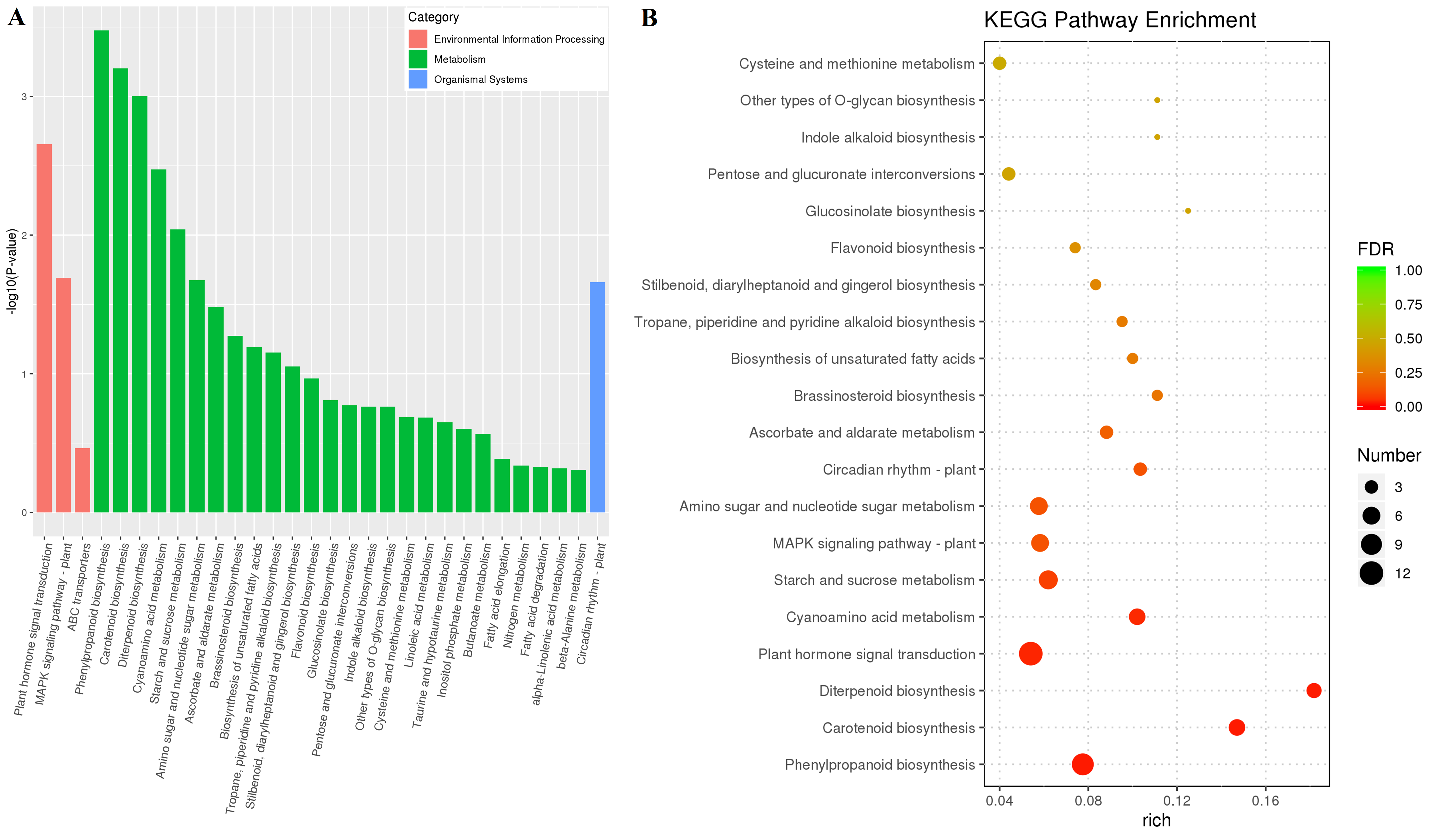

Supplement: Supplementary file 11 [file Image_11.tif]
